# Loss of RNF41 promotes bladder cancer metastasis through increasing NUDC stability to enhance tubulin polymerization

**DOI:** 10.1038/s41419-025-07758-y

**Published:** 2025-06-10

**Authors:** Fuchun Zheng, Sheng Li, Situ Xiong, Zhongqi Li, Ruize Yuan, Zhipeng Wang, Hao Wan, Jiahao Liu, Qianxi Dong, Xiaoqiang Liu, Wan Pang, Haibo Xi, Bin Fu, Songhui Xu

**Affiliations:** 1 Jiangxi Provincial Key Laboratory of Urinary System Diseases, Department of Urology, The First Affiliated Hospital, Jiangxi Medical College, Nanchang University, Nanchang, Jiangxi China; 2 College of Chemical and Environmental Engineering, Shanghai Institute of Technology, Shanghai, China

**Keywords:** Cancer, Biochemistry

## Abstract

Bladder cancer (BCa) is a representative of urological cancer with a high recurrence and metastasis is the leading cause of death from BCa. The underlying mechanism of BCa metastasis remains poorly defined. Here, we found RNF41 was significantly downregulated in BCa tissue and low level of RNF41 is associated with BCa progression and poor prognosis. RNF41 knockdown promoted cell migration and invasion in both in vitro and in tail vein lung metastasis model, while ectopic RNF41 expression exhibited the opposite effects. Mechanically, we revealed that RNF41 directly interacted with NUDC and ubiquitinates NUDC to promote its degradation. Clinically, RNF41 was significantly downregulated in metastatic BCa tissues and negatively associated with NUDC expression. Furthermore, we identified that RNF41 promoted BCa lung metastasis through NUDC regulated β-tubulin depolymerization. In summary, these findings support that RNF41 was a tumor suppressor in BCa metastasis and highlights that targeting RNF41-NUDC-β-tubulin axis could be a valuable strategy to ameliorate BCa progression and metastasis.

## Introduction

Bladder cancer (BCa) is the most prevalent malignancy within the genitourinary tract and stands as the sixth most prevalent cancer worldwide [1]. The majority of BCa cases are urothelial carcinomas, with approximately 75% classified as non-muscle-invasive BCa (NMIBC) and around 25% as muscle-invasive BCa (MIBC) [2]. Notably, the 5-year survival rate for patients with MIBC is considerably lower than that of NMIBC, estimated at 60–70% [3]. The most frequent sites for distant metastases in BCa include the lymph nodes, bones, lungs, and retroperitoneum, affecting approximately 50% of MIBC cases [4, 5]. Alarmingly, the median survival time for untreated patients with distant metastases is only 3–6 months [6]. Since the late 1980s, cisplatin-based neoadjuvant chemotherapy has been the established treatment for advanced BCa; however, it extends the median survival for metastatic cases to just about 14 months [7]. Given these challenges, metastatic BCa remains a significant public health concern, underscoring the urgent need for identifying novel metastasis-related gene therapy targets as a clinical priority.

Post-translational modifications (PTMs) are crucial for controlling protein functions and participate in diverse cellular processes by modulating protein subcellular localization and activity [8]. Ubiquitination is a form of PTM facilitated by ubiquitin ligases and deubiquitinases, which are vital for maintaining protein stability and functionality [9]. This modification is reversible, and an imbalance between ubiquitination and deubiquitination can result in irregular protein build-up or breakdown, potentially leading to cancer development [10]. RNF41 is an E3 ubiquitin ligase with a RING finger that includes four essential domains: a RING-finger region, B-box motif, coiled-coil segment, and a carboxy-terminal domain that engages with Epidermal growth factor receptor 3 (ErbB3) [11, 12]. Aberrations in RNF41 expression have been associated with several cancers, including those of the breast, colon, prostate, and liver [13–16]. Recent studies indicate that RNF41 plays a critical role in promoting cancer cell migration and invasion [17]. Qiu et al. found that RNF41-mediated degradation of ErbB3 can inhibit the activation of Phosphatidylinositol 3-Kinase/Protein Kinase B (PI3K/AKT), thereby suppressing the growth and survival of cancer cells [18]. Meanwhile, studies have shown that T-Box Transcription Factor 1 (TBX1) acts as a tumor suppressor in thyroid cancer by regulating its downstream target RNF41 to inhibit the activity of the PI3K/AKT pathways [19]. However, the precise mechanisms through which RNF41 influences the progression of BCa remain largely unexplored. Therefore, further investigation into the role of RNF41 as a potential therapeutic target in BCa is warranted.

Nuclear distribution protein C (NUDC) was initially discovered as a nuclear motility regulator in filamentous fungi [20]. Subsequent research has demonstrated that NUDC-associated motor proteins influence tubulin stability at axonal terminals [21]. NUDC can organize tubulin in the midbody and interact with motor proteins to form complexes that modulate various cellular processes, including mitosis and cell migration [22–24]. Tumor cell migration and invasion are crucial processes in the spread of cancer [25, 26]. Tubulin dynamics play a role in the formation and disassembly of adhesion complexes, which are essential for linking cells to the extracellular matrix during migration [27]. Additionally, tubulin organizing centers, such as centrosomes, help maintain directionality in polarized cell movement [28]. Tubulin are critical for tumor cell invasion and migration, profoundly impacting cellular functions in both health and disease [29]. Thus, targeting the NUDC/tubulin pathway may offer a promising strategy for inhibiting tumor cell migration, invasion, and metastasis.

In this study, we revealed that deletion of RNF41 promoted BCa cell migration and invasion. At the mechanistic level, RNF41 binds directly to NUDC, leading to its ubiquitination, which triggers tubulin reorganization and facilitates the migration and invasion of BCa cells. Low RNF41 expression showed a negative correlation with high NUDC levels, implying that RNF41 acts as a tumor suppressor in the metastasis of BCa. Targeting the RNF41/NUDC complex to induce tubulin reorganization could present a promising approach to hinder invasive BCa.

## Materials and methods

### Cell culture

Human BCa cell lines T24 and BIU, along with 293T, were sourced from ATCC. All cell lines have undergone recent authentication. BIU cells were cultured in RPMI-1640 medium (Gibco, China) supplemented with 10% fetal bovine serum (FBS). For T24 and 293T cells, Dulbecco’s modified Eagle’s medium (DMEM) (Gibco, China) with 10% FBS was utilized for culture.

### Histopathology

Conduct histopathological evaluation following the methodology was performed as previously [30]. Briefly, bladder tissue is preserved in 10% neutral buffered formalin and embedded in paraffin. The Clinical Medical Research Center of the First Affiliated Hospital of Nanchang University performed sectioning (5 µm), dewaxing, rehydration, and staining on paraffin-embedded specimens. Histological analysis of bladder tissue stained with HE and Ki67 was carried out by pathologists certified by the Board of Directors of the First Affiliated Hospital of Nanchang University.

### Immunohistochemistry (IHC)

The collection of tissue samples received approval from the Ethics and Internal Review Boards of the First Affiliated Hospital of Nanchang University. Bladder tissue microarrays were purchased from Spector Biotech Inc. (Shandong, China). Paraffin-embedded sections of human or mouse tumors underwent IHC staining. Slides were dewaxed, rehydrated with xylene, washed with gradient alcohol and PBS, and subjected to antigen retrieval. Endogenous peroxidase activity was blocked with TBS/H_2_O_2_, followed by overnight incubation with primary antibodies at 4 °C. The next day, slides were warmed to room temperature for 30 minutes, then incubated with secondary antibodies for another 30 min, stained with DAB, and counterstained with hematoxylin. RNF41 and NUDC expression was scored semiquantitatively: 0 (no staining), 1 (weak), 2 (moderate), and 3 (strong), with scores of 2 and 3 indicating high expression. The average score from two pathologists established the final immunostaining score, and the chi-square (χ²) test was applied to statistically analyze RNF41 and NUDC expression.

### Immunostaining

The slides were fixed with 4% paraformaldehyde for 15 min, rinsed with PBS, and immersed in 0.5% Triton X-100 for 10 min. The serum was blocked for 30 min at ambient temperature, then incubated with the primary antibody overnight at 4 °C. The next day, the cells were exposed to a fluorescently labeled secondary antibody for 1 h, and the nuclei were stained with DAPI. Observation was performed at ×400 or ×1000 magnification using a fluorescence microscope.

### BALB/c nude mice lung metastasis model

All BALB/c nude mice (4 weeks old) were sourced from Charles River Laboratories in Beijing, China. All animals involved in this research were treated humanely in accordance with relevant regulations, policies, and guidelines concerning animal welfare. The Institutional Animal Care and Use Committee of the First Affiliated Hospital of Nanchang University approved all experimental procedures involving animals. The indicated shControl, shRNF41, shNUDC, and or shRNF41+shNUDC T24 cells (1 × 10^6^) transduced with luciferase were injected into the mice via the tail vein (6 mice per group) to model tumor metastasis. Lung metastasis detection began on the 20th day post-injection. Mice received an injection of 5 mg/kg D-Luciferin (YEASEN, 40902ES02) and were imaged after 5 min using a LAGO imager with Aura imaging software (Spectral Instruments Imaging) at the Animal Imaging Facility of the First Affiliated Hospital of Nanchang University. Two weeks later, the mice were euthanized. Experienced researchers then quantified the number of pulmonary metastases through visual assessment, and intact tissues were processed for histological analysis as previously described.

### Statistical analysis

Kaplan–Meier (KM) survival curves were applied to assess OS among different patient cohorts. Data visualization, chart generation, and statistical analyses were conducted using GraphPad Prism software (version 8.0). Variations in qRT-PCR outcomes, lung metastasis model features, and cellular behaviors (including migration, invasion, proliferation, among others) between groups were evaluated through a t-test. Relationships were examined using Pearson’s correlation coefficient. Quantitative results are presented as the standard deviation (SD) unless otherwise noted. Statistical significance is indicated by (^*^P < 0.05; ^**^P < 0.01). All procedures were conducted in triplicate. Additional methods and materials can be found in the Supplemental Information.

## Results

### RNF41 is lower expressed in BCa tumor and is associated with BCa progression

**Fig. 1:**
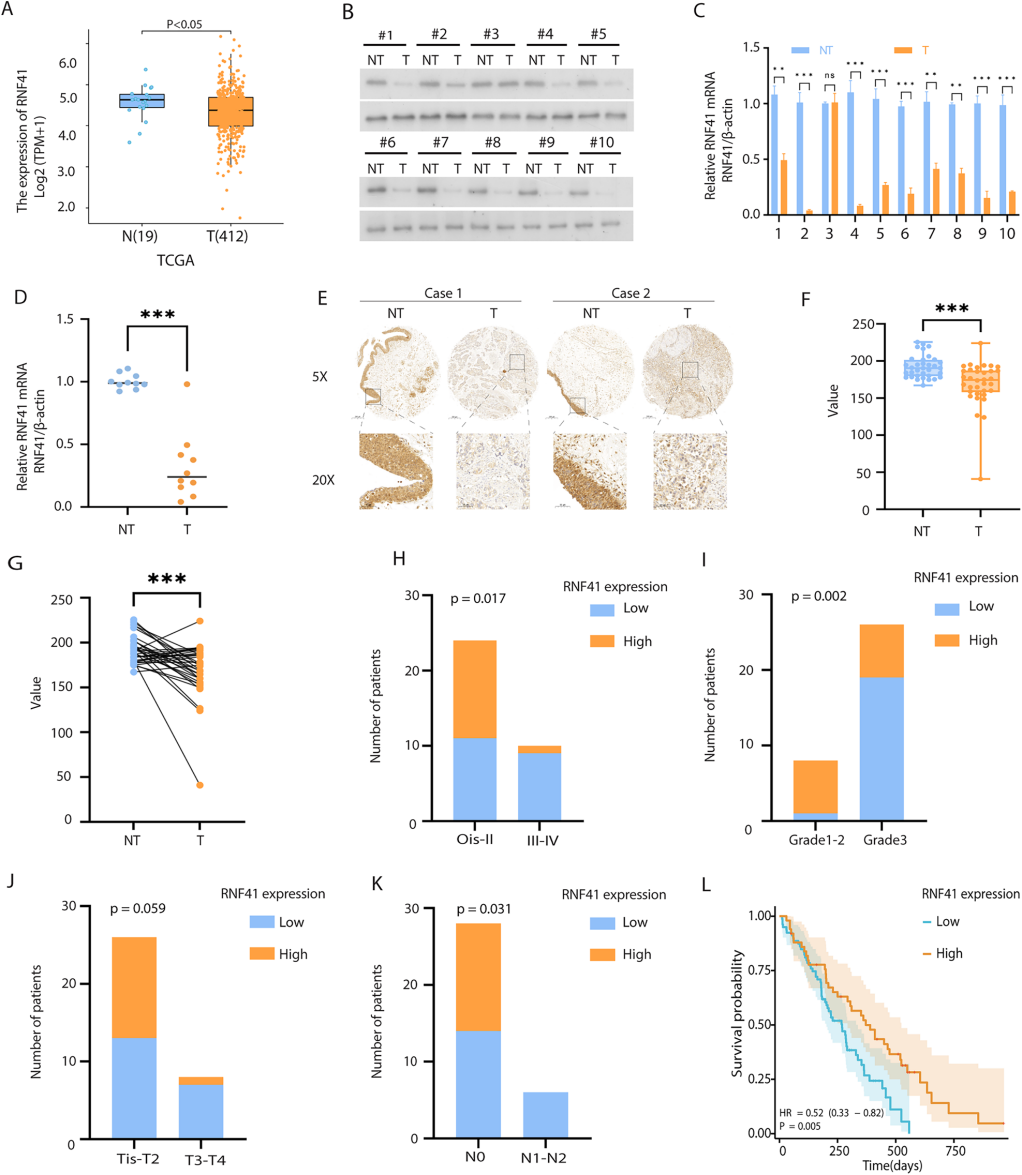
Downregulation of RNF41 in bladder cancer (BCa). **A** RNF41 expression in normal tissues (*n* = 19) vs. BCa tissues (*n* = 412) from TCGA database. **B** Western blot showing RNF41 protein levels in 10 fresh BCa tissues (T) and matched paracancerous tissues (NT). **C**, **D** Real-time PCR analysis of RNF41 mRNA expression in 10 fresh BCa tissues and matched paracancerous tissues. **E** Representative immunohistochemistry (IHC) images showing RNF41 protein expression in BCa and adjacent tissues using tissue chips. Scale bar, 50 μm. **F**, **G** RNF41 protein downregulation in adjacent non-tumor tissues and BCa tissues (**F**) and in paired adjacent and matched BCa tissues (**G**). **H**–**K** Correlation of low RNF41 expression with higher stage (*p* = 0.017), grade (*p* = 0.002), clinical T3/T4 (*p* = 0.059), and N1 (*p* = 0.031). **L** Overall survival analysis in 129 BCa patients, showing significantly shorter survival in those with low RNF41 expression (*p* = 0.005). Data provided by The First Affiliated Hospital of Nanchang University.

To investigate and confirm RNF41 expression levels in BCa, we initially examined RNF41 expression in normal bladder tissue versus BCa tissue using the Cancer Genome Atlas (TCGA) database. This analysis revealed that RNF41 expression was lower in BCa tissue compared to normal tissue (Figs. 1A and S1A). Western blot analysis (Fig. 1B) and real-time PCR (Fig. 1C, D) further demonstrated that both RNF41 protein and mRNA levels were reduced in fresh BCa samples (*n* = 10) relative to paired adjacent non-cancerous tissues (*n* = 10). IHC staining on tissue microarrays confirmed decreased RNF41 protein expression in BCa tissues compared to adjacent tissues (Fig. 1E). Based on RNF41 IHC staining intensity, patients were classified into low (*n* = 20) and high (*n* = 14) RNF41 expression groups (Fig. 1F, G). Among the 34 BCa specimens, 20 exhibited low RNF41 expression, whereas only 14 showed high RNF41 levels (Table S1). Notably, low RNF41 expression was correlated with lymph node metastasis and poorer histological grade, but not associated with age, gender, or T stage (Fig. 1H–K and Table S1). KM analysis demonstrated that patients exhibiting elevated RNF41 expression showed better OS compared to those with reduced RNF41 levels (Fig. 1L). These findings suggest that RNF41 expression is downregulated in BCa and that higher RNF41 protein levels may be indicative of a more favorable prognosis.

### Loss of RNF41 promotes BCa cell migration and invasion in vitro and vivo

**Fig. 2:**
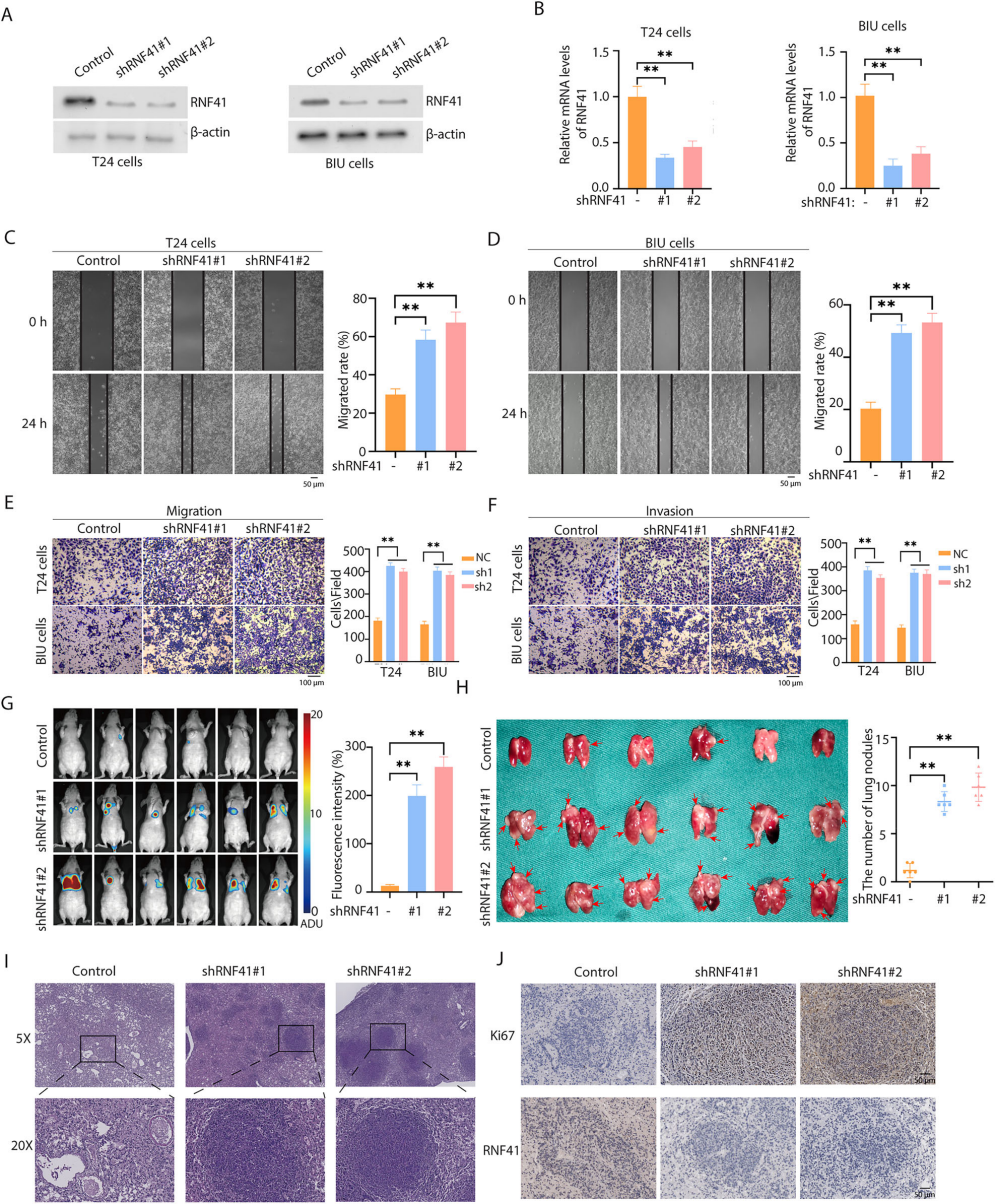
Inhibition of RNF41 promotes migration of BCa cells. **A**, **B** Knockdown efficiency of RNF41 by shRNA, measured via western blot and quantitative RT-PCR (**p < 0.01). **C**, **D** Migration abilities of T24 and BIU cells after RNF41 knockdown assessed using a wound healing assay (**p < 0.01)^.^ Scale bar, 50 μm. **E**, **F** Representative images and quantification of migrated or invading cells in a transwell assay, both without and with matrix penetration (**p < 0.01). Scale bar^,^ 100 μm. **G** Fluorescence imaging of experimental nude mice injected with T24-Luc cells, comparing fluorescence intensity (**p < 0.01). **H** Lung imaging and quantification of pulmonary nodules (**p < 0.01). **I** Hematoxylin and eosin (HE) staining of lungs with metastatic tumors. **J** Ki67 and RNF41 immunohistochemistry (IHC) staining of lungs with metastatic tumors. Statistical analyses were performed using ANOVA. *p < 0.05; **p < 0.01. Scale bar, 100 μm.

To elucidate the function of RNF41 in BCa, we used two distinct RNF41 shRNAs to knock down its expression in T24 and BIU cell lines via lentiviral transduction and also generated an RNF41-overexpression plasmid to upregulate RNF41 in BCa cells. Western blot and qRT-PCR confirmed the efficiency of both knockdown and overexpression of RNF41 at protein and mRNA levels in both cell lines (Figs. 2A, B and S2A, B). Wound-healing assays demonstrated that RNF41 knockdown markedly increased BCa cell migration in vitro (Fig. 2C, D). Transwell migration and invasion assays further validated that RNF41 depletion enhanced both migratory and invasive capacities of BCa cells (Fig. 2E, F). To examine the role of RNF41 in metastasis in vivo, we used a tail vein injection model for BCa lung metastasis by injecting T24-Luciferase shControl or RNF41-knockdown cells into nude mice. Bioluminescence imaging (BLI) showed an increase in lung metastases in the RNF41 knockdown group (Fig. 2G). Macroscopic examination of lung surfaces revealed more visible nodules in RNF41-deficient mice (Fig. 2H), and HE staining confirmed a significantly higher number of metastases (Fig. 2I), with an increase in Ki67-positive cells indicating higher proliferative activity in metastatic lesions (Fig. 2J). Notably, RNF41 knockdown or overexpression did not alter BCa cell proliferation in EDU assays (Figs. S1B, C and S2G, H) or in colony formation assays (Figs. S1D, E and S2I, J). However, RNF41 overexpression did decrease BCa cell migration and invasion (Fig. S2C–F). Collectively, these data suggest that RNF41 depletion promotes BCa cell metastatic behavior both in vitro and in vivo, while its overexpression curtails such aggressive traits.

### RNF41 interacts with NUDC and facilitates its degradation via the ubiquitin-proteasome pathway

**Fig. 3:**
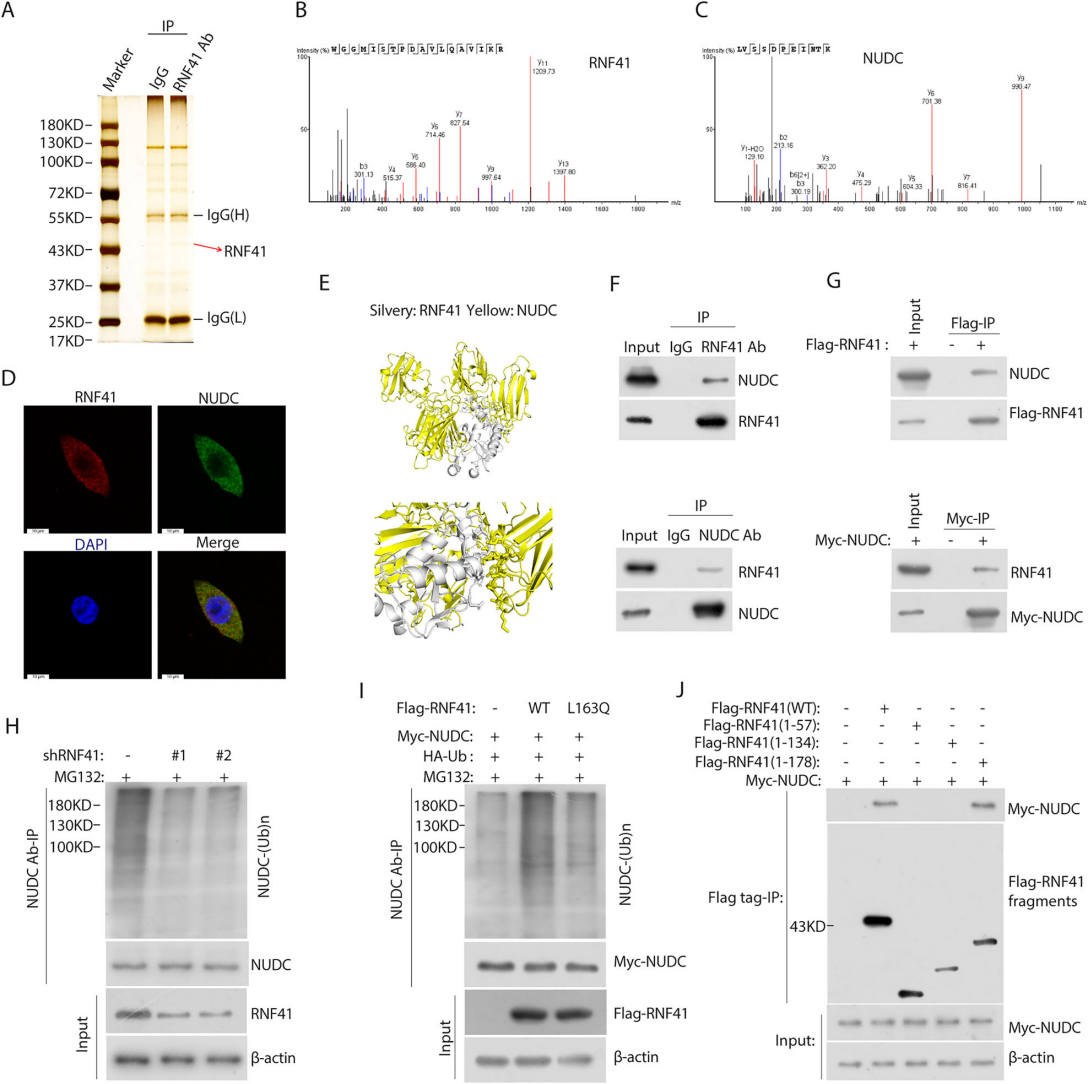
RNF41 directly interacts with and ubiquitinates NUDC. **A** Proteins interacting with RNF41 were detected using co-immunoprecipitation (co-IP) and silver staining in T24 cells. **B**, **C** Peptide fragment mass spectrum for RNF41 and NUDC protein. **D** Fluorescence microscopy revealed co-localization of RNF41 (red) and NUDC (green) in T24 cells, with nuclear staining by 4’, 6-diamidino-2-phenylindole (DAPI; blue). Scale bar, 10 μm. **E** Protein docking map illustrating the predicted interaction between RNF41 and NUDC. **F** Endogenous co-immunoprecipitation results showing the interaction of RNF41 and NUDC in T24 cells. **G** Co-immunoprecipitation of FLAG-RNF41 and MYC-NUDC in T24 cells, transfected with FLAG-RNF41 and MYC-NUDC plasmids for 36 h. **H** HEK293T cells co-transfected with the indicated shRNA underwent immunoprecipitation (IP) with anti-NUDC antibody, followed by immunoblotting (IB) with antibodies against NUDC, RNF41, and β-actin. Cells were treated with 20 μM MG132 for 6 h prior to harvesting. **I** HEK293T cells co-transfected with Myc-NUDC, HA-ubiquitin (HA-Ub), and Flag-tagged RNF41 WT or RNF41 L163Q, with lysates subjected to IP using anti-NUDC antibody followed by IB with antibodies against Myc-tagged, Flag-tagged proteins, and β-actin. Cells were treated with 20 μM MG132 for 6 h before collection. **J** HEK293T cells were co-transfected with Myc-NUDC and Flag-tagged full-length RNF41 or its deletion mutants, followed by immunoprecipitation with Flag beads and immunoblotting with antibodies against Myc and Flag.

To clarify the molecular mechanism underlying RNF41-mediated progression of BCa, we conducted immunoaffinity purification followed by mass spectrometry (MS) analysis to identify RNF41-interacting proteins (Fig. 3A). The MS results (Fig. 3B, C) showed that RNF41 interacted with NUDC, a nuclear motor protein. And we confirmed by following experiments. First, immunofluorescence staining demonstrated that endogenous RNF41 and NUDC were primarily co-localized in the cytoplasm of T24 and BIU cell lines (Figs. 3D and S3A). Second, protein docking analysis indicated a potential interface for protein-protein interaction between RNF41 and NUDC (Fig. 3E). Third, co-immunoprecipitation experiments showed that endogenous RNF41 co-precipitated with NUDC in T24 cells (Fig. 3F), while FLAG-tagged RNF41 and MYC-tagged NUDC were able to interact with their respective endogenous counterparts in T24 cells (Fig. 3G). The ubiquitin-proteasome system is a well-known mechanism that regulates protein stability. Our findings indicated that silencing RNF41 led to decreased polyubiquitination of NUDC (Fig. 3H). Moreover, overexpression of wild-type RNF41, as opposed to the L163Q mutant (where L163 is the active site of the RNF41 enzyme), resulted in increased polyubiquitination of NUDC (Fig. 3I). RNF41 consists of four domains: the ring finger domain (amino acids 1-57), the B-box domain (two TRAF-like zinc fingers; amino acids 58–134), the coiled-coil domain (amino acids 135-178), and the carboxy-terminal ErbB3 interaction region (amino acids 179–317) [11, 12]. We conducted co-immunoprecipitation assays utilizing truncated variants of RNF41, confirming an interaction between the coiled-coil domains of NUDC and RNF41 (Fig. 3J).

### RNF41 modulates NUDC stability and exhibits a negative correlation with NUDC in BCa samples

**Fig. 4:**
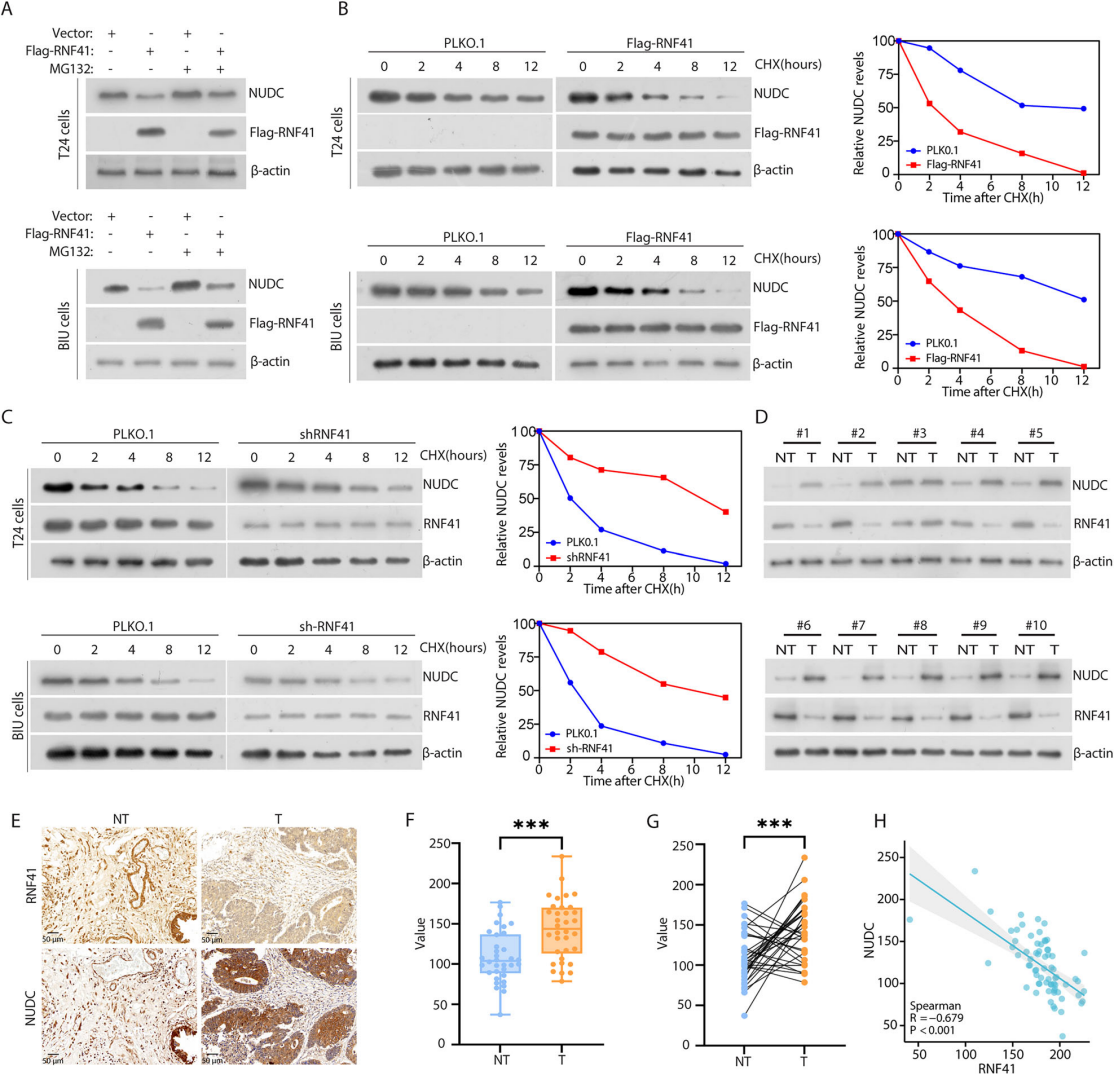
RNF41 regulates the stability of NUDC. **A** T24 and BIU cells transfected with the specified constructs were treated with or without MG132 for 6 h before collection. RNF41 and NUDC proteins were analyzed using the indicated antibodies. **B** Left: FLAG-RNF41 plasmid was introduced into T24 and BIU cells, followed by the addition of 50 μg/ml cycloheximide (CHX) at 0, 2, 4, 8, and 12 h. Cells were then harvested, and western blot analysis with NUDC antibodies determined the half-life of NUDC protein. Right: Quantification of NUDC protein levels. **C** Left: RNF41 shRNA was transfected into T24 and BIU cells for 36 h, followed by treatment with 50 μg/ml CHX at specified intervals (0, 2, 4, 8, 12 h) before collection. The half-life of NUDC protein was assessed via western blot. Right: Quantification of NUDC protein levels. **D** Expression analysis of RNF41 and NUDC in 10 freshly paired metastatic BCa tissue samples (T) and matched adjacent non-tumor tissues (NT). β-actin served as the loading control. **E** Representative staining images of RNF41 and NUDC in BCa tissues. Scale bar, 50 μm. **F**, **G** Compared to adjacent tissues, NUDC protein levels were elevated in BCa tissues (**F**), and in paired samples, NUDC was also found to be upregulated in BCa tissues (**G**). **H** Correlation analysis between RNF41 and NUDC expression levels in BCa tissues, with statistical analyses performed using the χ² test (*P* < 0.001). R indicates Pearson’s correlation coefficient.

To explore how RNF41 stabilizes NUDC, we treated specific BCa cell lines with or without the proteasome inhibitor MG132 and assessed protein expression levels. Our findings indicated that MG132 partially mitigated RNF41-mediated degradation of NUDC (Fig. 4A). Additionally, cycloheximide (CHX) treatment revealed that overexpression of RNF41 notably expedited the breakdown of NUDC proteins (Fig. 4B), while depletion of RNF41 resulted in an extended half-life of NUDC (Fig. 4C). To further validate that RNF41 enhances ubiquitination of NUDC in BCa samples, we examined the expression levels of RNF41 and NUDC in BCa tissues. We observed that lower levels of RNF41 protein levels were associated with higher NUDC expression in BCa tissues (Fig. 4D). IHC staining of RNF41 and NUDC in BCa tissues demonstrated that diminished RNF41 expression was negatively associated with elevated NUDC levels (Fig. 4E). Among the 34 BCa samples analyzed, 22 exhibited strong NUDC expression, while 20 displayed low RNF41 expression (Fig. 4F, G). Pearson correlation analysis demonstrated a strong inverse relationship (*R* = -0.675, *P* < 0.001) between the expression levels of RNF41 and NUDC (Fig. 4H). Furthermore, high NUDC expression was linked to larger tumor size but showed no correlation with age, histological grade, or lymph node metastasis (Table S2). In summary, these findings indicate that RNF41 is downregulated in BCa, negatively correlates with NUDC expression, and targets NUDC for ubiquitination and modulation of stability.

### RNF41 regulates NUDC-induced tubulin depolymerization

**Fig. 5:**
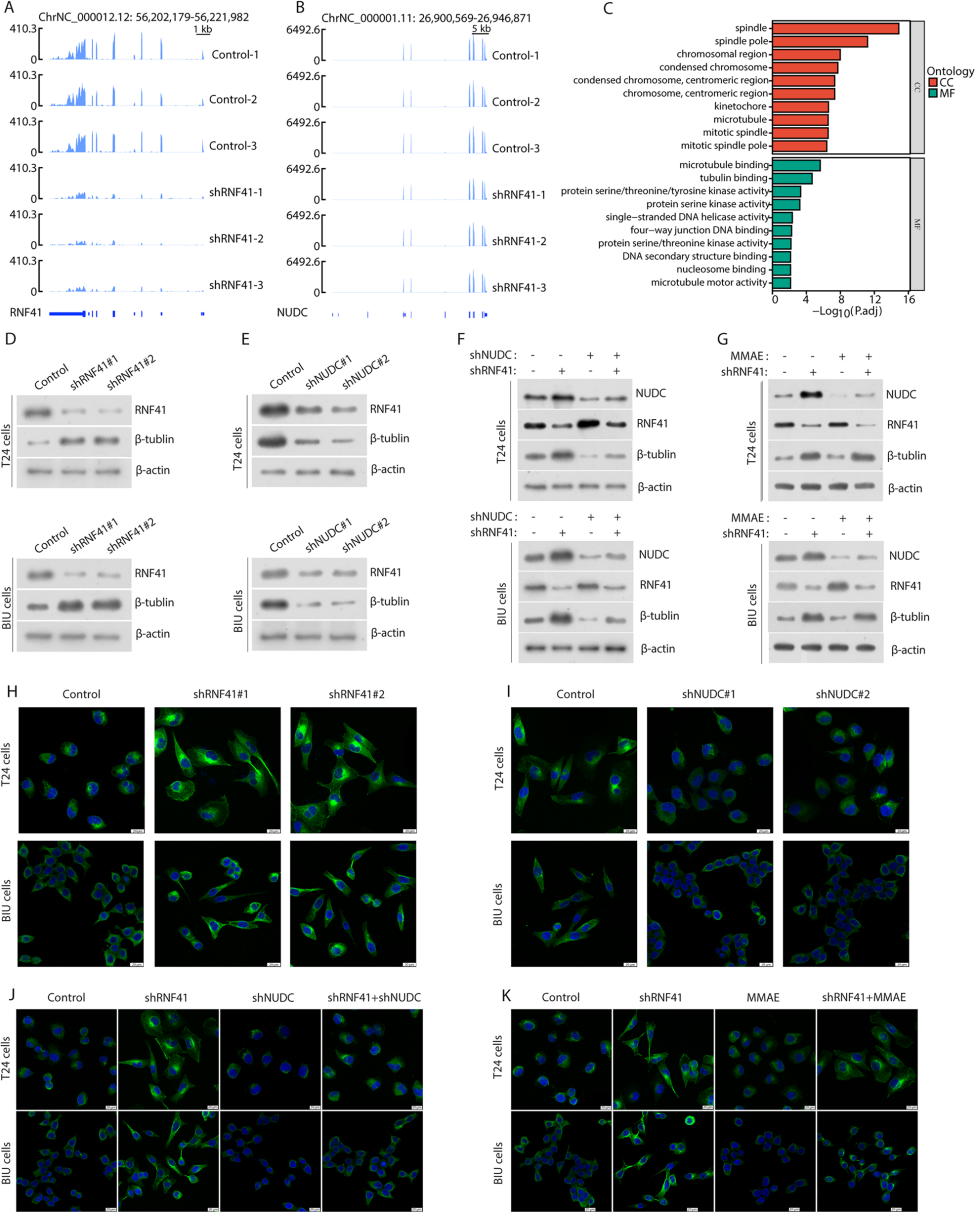
RNF41 regulates NUDC-induced tubulin depolymerization. Sequencing peaks of RNF41 (**B**) and NUDC (**A**) after RNF41 knockdown. **C** Gene Ontology (GO) analysis of differentially expressed genes following RNF41 knockdown in T24 cells. CC represents cellular component; MF denotes molecular function. **D** Western blot analysis was performed to assess protein levels in T24 and BIU cells stably expressing RNF41 shRNA. **E** Western blot was conducted on T24 and BIU cells stably expressing NUDC shRNA to evaluate the indicated protein levels. **F** T24 and BIU cells stably expressing either NUDC or RNF41 shRNA plasmids, with or without NUDC shRNA, underwent western blot analysis to measure specific protein levels. **G** T24 and BIU cells with stable RNF41 shRNA expression were treated with or without Monomethyl auristatin E (MMAE) (5 μM) and subsequently analyzed by western blot for the specified proteins. **H** Confocal microscopy images of T24 and BIU cells stably expressing Control or RNF41 shRNA. **I** Confocal microscopy images of T24 and BIU cells stably expressing Control or NUDC shRNA. **J** Confocal images of T24 and BIU cells stably expressing Control, RNF41 shRNA, and/or NUDC shRNA. **K** Confocal images of T24 and BIU cells stably expressing Control, RNF41 shRNA, and/or MMAE. Tubulin (green) was visualized using β-tubulin antibodies, while DNA (blue) was stained with DAPI. Scale bar, 20 μm.

To determine global gene regulation by RNF41 and identify how it regulates metastasis of BCa cells, we performed an RNA sequencing analysis of T24 cells expressing RNF41 shRNA. Sequencing data confirmed a reduction in RNF41 transcription levels after its knockdown (Fig. 5A), while NUDC transcription remained unaffected (Fig. 5B). Volcano plot (Fig. S4A) and cluster heatmap (Fig. S4B) analysis indicated that RNF41 knockdown led to the upregulation of 979 genes and downregulation of 1045 genes. Gene Ontology (GO) enrichment analysis revealed that RNF41 regulated genes, which were associated with tubulin function (Fig. 5C) and considering that NUDC is associated with tubulin motor proteins, which are essential for the intracellular cytoskeleton framework and facilitate tumor metastasis by influencing cell division and migration [31, 32]. These suggest that RNF41-NUDC complex may regulate metastasis of BCa cells through tubulin. To determine whether RNF41 enhances BCa cell metastasis via NUDC-induced tubulin depolymerization, we first stably transfected cells with RNF41 shRNA or NUDC shRNA plasmids. We observed that knocking down RNF41 elevated the protein levels of β-tubulin in both T24 and BIU cell lines (Fig. 5D), while silencing NUDC led to decreased β-tubulin levels (Fig. 5E). Furthermore, in T24 and BIU cells with RNF41 knockdown, silencing NUDC significantly reversed the effects of RNF41 depletion on β-tubulin levels (Fig. 5F). To further demonstrate the impact of RNF41 on tubulin, we selected the tubulin inhibitor Monomethyl auristatin E (MMAE). We first conducted half maximal inhibitory concentration (IC50) assays in T24 and BIU cells treated with MMAE. The results indicated that the IC50 values for both cell lines ranged from 5 to 10 μM (Fig. S3B). Subsequently, we employed flow cytometry to examine the cell cycle of T24 and BIU cells treated with 5 μM MMAE. The findings revealed that MMAE significantly inhibited the G2/M phase (Fig. S3C–F). Ultimately, the addition of MMAE to RNF41-knockout cells also significantly reversed the impact of RNF41 depletion on β -tubulin levels (Fig. 5G). To further assess the stability of RNF41 and NUDC on tubulin networks, we conducted β-tubulin immunofluorescence analysis on cells stably expressing RNF41 shRNA or NUDC shRNA, with or without NUDC shRNA. Compared to control cells, RNF41 knockdown cells exhibited no significant tubulin depolymerization, but displayed notable cell elongation and filamentous pseudopodia, indicating enhanced tubulin network stability (Fig. 5H). In contrast, the NUDC knockdown cells showed complete tubulin depolymerization (Fig. 5I). Additionally, silencing NUDC in RNF41 knockdown cells restored the inhibitory effects of RNF41 (Fig. 5J), which was also observed with MMAE treatment in RNF41 knockdown cells (Fig. 5K).

### RNF41 promotes BCa cell metastasis in a NUDC-dependent manner

**Fig. 6:**
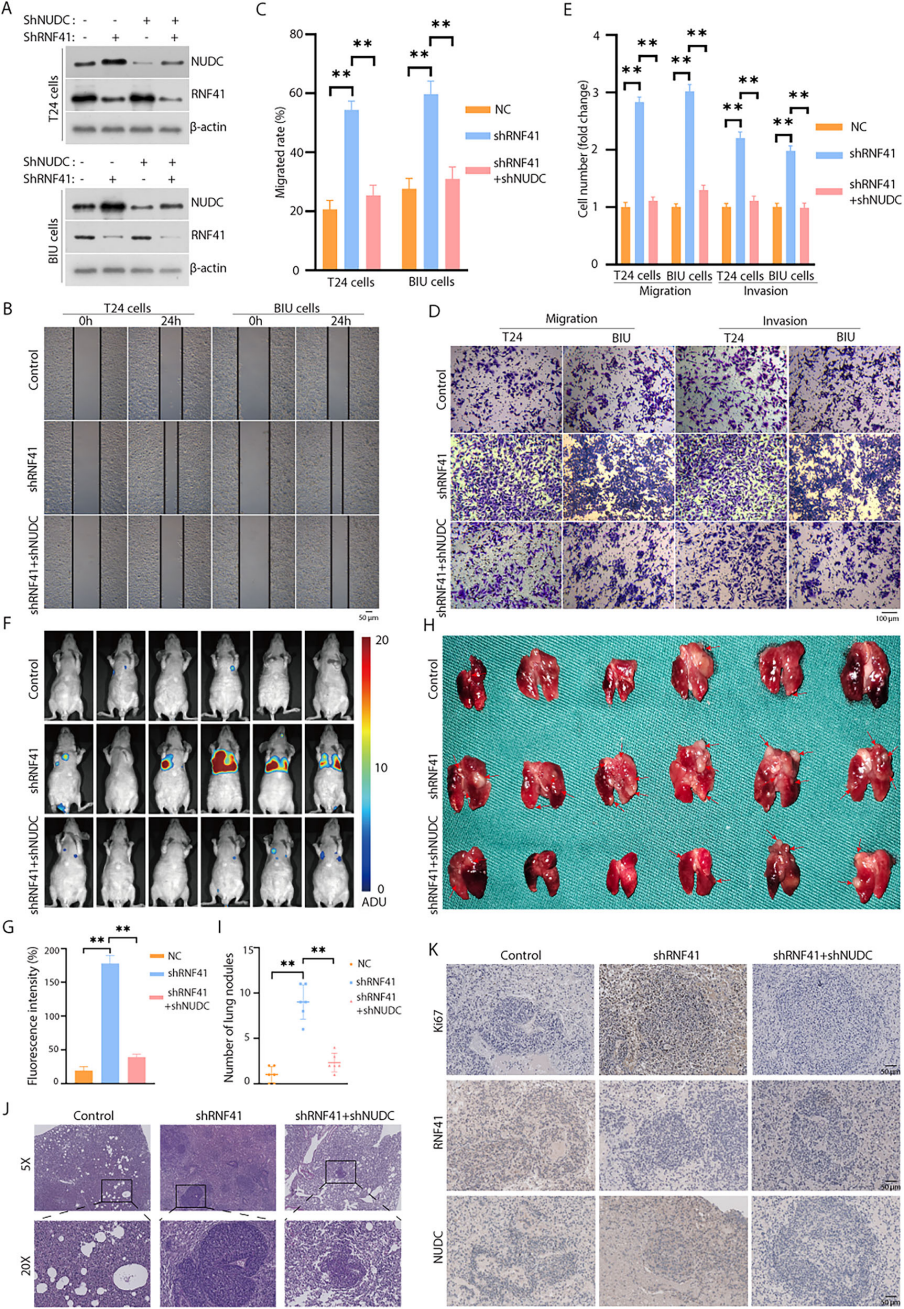
NUDC knockdown rescue the enhanced migratory and invasive abilities caused by RNF41 knockdown, both in vitro and in vivo. **A** The effectiveness of RNF41 and NUDC knockdown was assessed using western blot analysis. **B**, **C** Wound healing assays were conducted to evaluate the migratory potential of BCa cells, with migration rates calculated (**p < 0.01). Scale bar, 50 μm. **D**, **E** Representative images and quantification of migrated or invading cells from a transwell assay, conducted both with and without matrix penetration (**p < 0.01)^.^ Scale bar, 100 μm. **F**, **G** Fluorescence imaging of experimental nude mice injected with T24-Luc cells, comparing fluorescence intensity (**p < 0.01). **H**, **I** Lung imaging and quantification of pulmonary nodules (**p < 0.01). **J** Hematoxylin and eosin (HE) staining of lung tissue containing metastatic tumors. **K** Immunohistochemical staining for Ki67, RNF41, and NUDC in lung tissues with metastatic tumors. Scale bar, 50 μm.

**Fig. 7:**
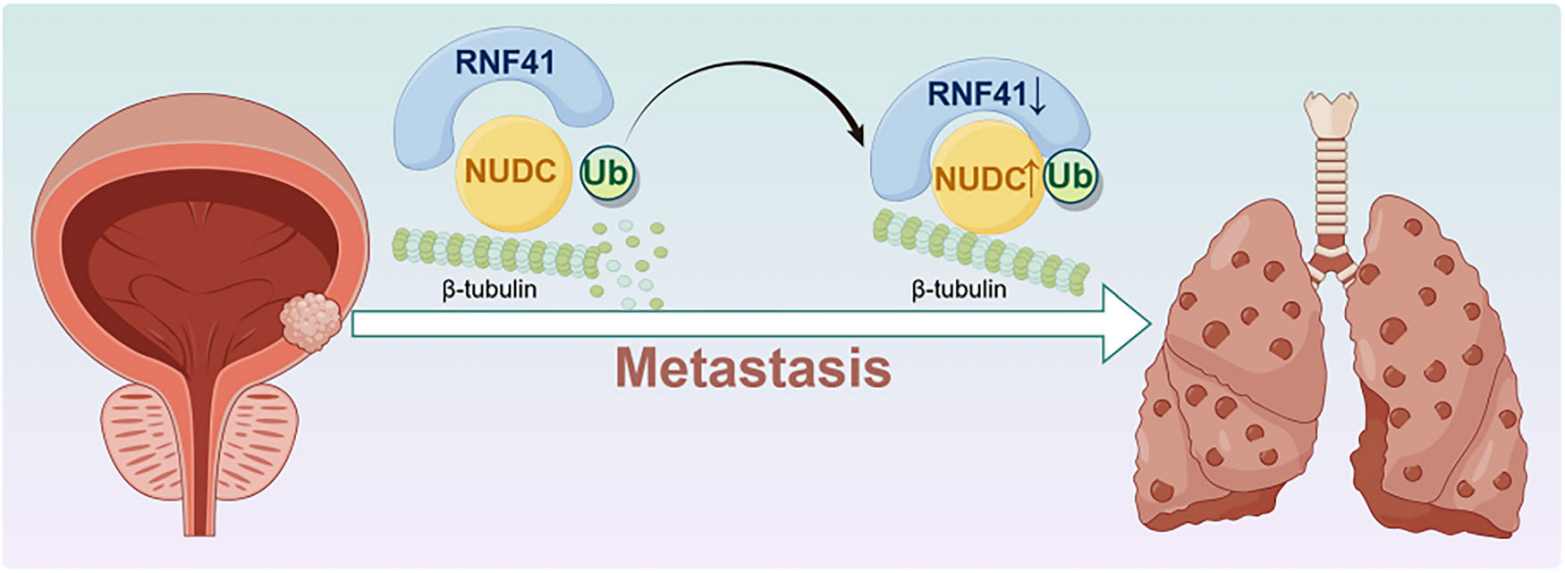
RNF41 induces canonical polyubiquitination of NUDC to enhance its stability and induce β-tubulin remodeling, which promotes BCa metastasis. Created with figdraw.com.

To clarify the specific roles of NUDC and RNF41 in BCa and to assess whether NUDC is essential for RNF41-mediated tumor metastasis, we generated T24 and BIU cells stably transfected with lentivirus expressing shRNF41 and shNUDC (Fig. 6A). The wound healing assay and transwell assays demonstrated that inhibition of RNF41 enhanced the migration and invasion of T24 and BIU cells, while silencing NUDC in conjunction with RNF41 inhibition significantly rescued the effects of RNF41 knockdown (Fig. 6B–E). While, inhibition of RNF41 or NUDC did not affect the proliferation of T24 and BIU cells by the EDU assay (Fig. S4C, D) and the colony formation assay (Fig. S4E, F). Furthermore, to investigate the metastatic capabilities of NUDC and RNF41 in vivo, we established stable cell lines of luciferase shControl, luciferase shRNF41, and luciferase shRNF41 + shNUDC. These three cell lines were administered via intravenous injection into the tail veins of nude mice. After 20 days, we assessed the metastatic potential by measuring lung fluorescence. Compared to the control group, shRNF41 markedly enhanced in vivo migration while silencing NUDC could rescue this effect (Fig. 6F–I). The HE staining and IHC staining of Ki67, RNF41 and NUDC confirmed this phenomenon (Fig. 6J, K). In summary, these findings indicate that RNF41 facilitates BCa cell metastasis through a mechanism reliant on NUDC (Fig. 7).

## Discussion

Various E3 ubiquitin ligases are crucial in tumor initiation, progression, prognosis, and resistance to chemotherapy via the ubiquitin-proteasome system, positioning them as promising targets for cancer therapy [33, 34]. BCa is a prevalent malignancy that substantially affects patients’ quality of life [3]. Creating prognostic models that predict patient survival using key biomarkers offers a promising path to enhance BCa treatment precision. RNF41, an E3 ubiquitin ligase, is integral in regulating scaffold protein localization, cancer cell stemness, migration and invasion, cellular adhesion, tight junctions, and cell-matrix interactions [14, 35, 36]. Reduced RNF41 expression is linked to a poor prognosis in glioblastoma multiforme (GBM), with studies showing that restoring RNF41 levels decreases GBM cell migration and invasiveness [37, 38]. Additionally, RNF41-mediated degradation of ErbB3 impedes breast cancer cell growth and motility [13]. In pancreatic cancer, the retinoic acid derivative ABPN (A4-amino-2-(butyrylamino)phenyl(2E,4E,6E,8E)-3,7-dimethyl-9-(2,6,6-trimethyl-1-cyclohexenyl)-2,4,6,8-nonatetraenoate) demonstrates strong anti-cancer effects by upregulating RNF41 expression [39]. Elevated RNF41 expression is associated with metastatic prostate cancer, and silencing RNF41 impairs the growth, migration, and invasion of prostate cancer cells both in vitro and in vivo [15]. Additionally, RNF41 has been proposed as a potential prognostic biomarker and therapeutic target in hepatocellular carcinoma, with its expression positively correlating with the programmed cell death index [40]. These findings underscore the clinical significance of RNF41 and its promise as a therapeutic target in various malignancies. However, RNF41’s specific roles in tumorigenesis and especially in metastasis remain largely unexplored. In this study, we examine RNF41’s role in BCa, aligning with previous findings [14, 41]. Protein and transcriptional analyses confirmed that RNF41 is downregulated in BCa tissues. Wound healing assay, Transwell assays, and lung metastasis models demonstrated that RNF41 suppression significantly enhances BCa cell metastasis while having no significant effect on cell proliferation.

Immunoprecipitation combined with MS analysis revealed NUDC as an interacting protein with RNF41. NUDC’s expression and function are modulated by PTMs [42]. Among these, ubiquitination—strongly linked to protein stability and degradation—has garnered significant interest in cancer studies. NUDC plays a role in recruiting Plk1 to the centromere and ensuring proper tubulin attachment during early mitosis [43]. This function is essential for organizing stable central structures and completing cytokinesis. Research indicates that NUDC regulates actin dynamics and cell migration by stabilizing filaggrin 1 independently of Hsp90 [44]. Given that cell migration and invasion are critical steps in metastasis [45, 46], our study found that RNF41 influences NUDC stability by promoting its ubiquitin-proteasome pathway, facilitating BCa lung metastasis.

Tubulin are essential components of the intracellular cytoskeleton, involved in cell division, migration, signal transduction, and intracellular transport, making them significant in cancer cell metastasis [32]. In our research, the GO analysis following RNF41 knockdown revealed enrichment in tubulin-related pathways. Given NUDC’s close association with tubulin, RNF41 may influence tubulin depolymerization via NUDC. MMAE is a potent tubulin inhibitor widely used as a payload in antibody-drug conjugates (ADCs), such as Envolumab vedotin for advanced urothelial carcinoma [47]. After binding to target antigens on cancer cell surfaces, ADCs are internalized through endocytosis, where they interact with tubulin proteins. MMAE disrupts tubulin formation and interferes with mitotic spindle assembly [48]. In our study, we found that the MMAE treatment group exhibited greater tubulin depolymerization compared to the control group, which partially restored the effects of shRNF41 on tubulin levels. Overall, our encouraging preclinical findings underscore the potential for future clinical trials assessing RNF41 inhibitors as a therapeutic strategy for metastatic BCa. However, several limitations of this study warrant cautious interpretation. Firstly, the relatively small sample size may limit the statistical power and generalizability of our findings. Secondly, although we employed complementary in vitro and in vivo models to validate RNF41’s role, these systems cannot fully recapitulate the intricate tumor-stroma interactions and heterogeneity observed in human BCa microenvironments. Notably, the animal models may lack critical immune components and stromal cell diversity that modulate cancer metastasis in patients. Thirdly, our mechanistic exploration focused primarily on the RNF41-NUDC-tubulin axis, while post-translational modifications (e.g., phosphorylation or acetylation) of RNF41 and its interacting partners remain uncharacterized. Future studies employing advanced techniques such as mass spectrometry-based proteomics or CRISPR-based screening could elucidate these regulatory layers.

Despite these limitations, our study demonstrates that the loss of RNF41 fosters the growth and metastasis of BCa cells through a ubiquitination-dependent mechanism. As an E3 ubiquitin ligase, RNF41 influences the ubiquitination of NUDC, enhancing its stability and facilitating tubulin depolymerization, which in turn promotes lung metastasis of BCa. Clinically, reduced levels of RNF41 are negatively correlated with NUDC expression in BCa. Furthermore, our findings suggest that RNF41 may function as a new biomarker and a promising therapeutic target for metastatic BCa.

## Supplementary information

Supplementary Materials

original western blots

## Data Availability

The datasets used and/or analyzed during the current study are available from the corresponding author on reasonable request.
