# Supplementary material for: Loss of RNF41 promotes bladder cancer metastasis through increasing NUDC stability to enhance tubulin polymerization
Source: Cell Death Dis. 2025 Jun 10;16(1):443. doi: 10.1038/s41419-025-07758-y (PMC12152122; doi:10.1038/s41419-025-07758-y)
Supplement: Supplementary file 1 — Supplementary Materials [file 41419_2025_7758_MOESM1_ESM.doc]

**Supplementary information**

**Supplemental Figures, Tables, Methods and References**

**Supplementary Figures**

**Fig. S1**

**
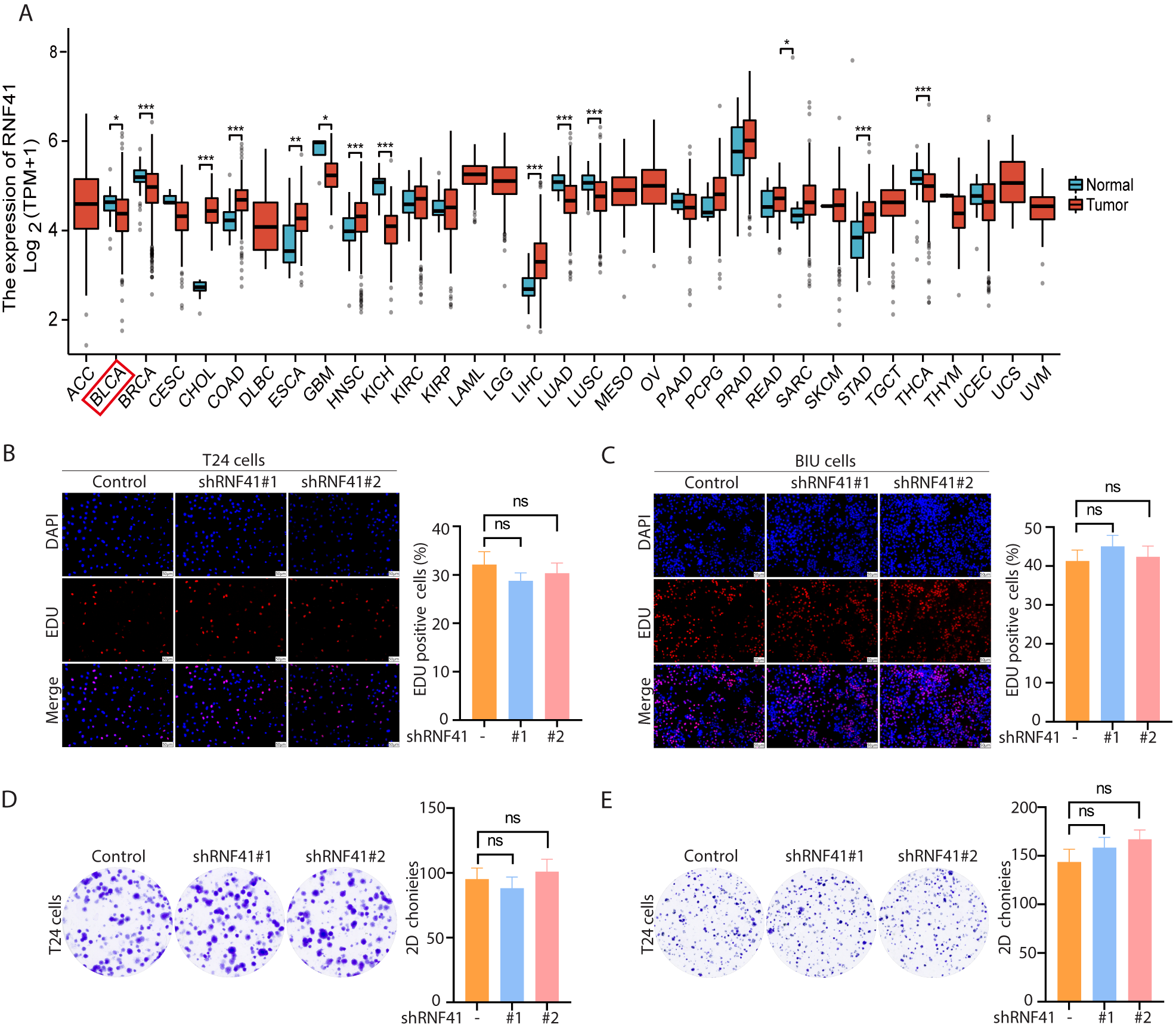
**

**Fig. S1**

(A) The RNF41 gene expression across 33 tumor types and normal tissues in the TCGA database (https://portal.gdc.cancer.gov/). (B) (C) Illustrative micrographs (left side) and corresponding quantification (right side) of Edu labeling in T24 or BIU cells stably transfected with either the lentiviral control plasmid or shRNF41. (D) (E) Colony formation assays demonstrated the impact of RNF41 knockdown on the survival of T24 and BIU cells post-transfection.

**Fig. S2**

**
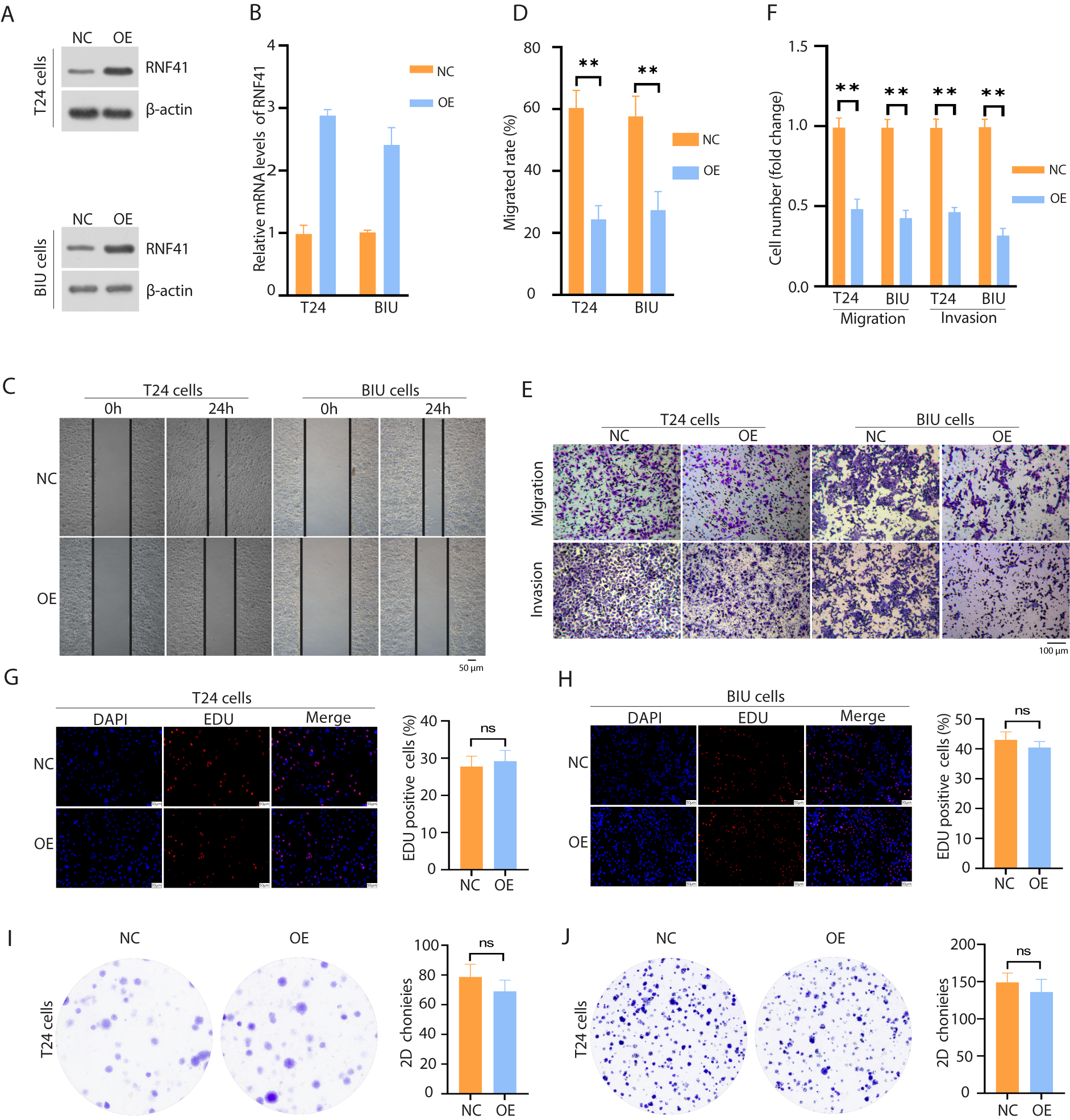
**

**Fig. S2**

(A) (B) The overexpression efficiency of RNF41, evaluated through western blotting and qRT-PCR (^**^*p* < 0.01). (C) (D) Migration capacity of T24 and BIU cells following RNF41 overexpression was measured using a wound healing assay (^**^*p* < 0.01). (E) (F) Representative images and quantification of migrating or invading cells in a transwell migration assay, both with and without matrix penetration (^**^*p* < 0.01). (G) (H) Representative micrographs (left) and quantification (right) of Edu labeling in T24 and BIU cells with RNF41 overexpression. (I) (J) Colony formation assays illustrated the effect of RNF41 overexpression on the viability of T24 and BIU cells post-transfection.

**Fig. S3**


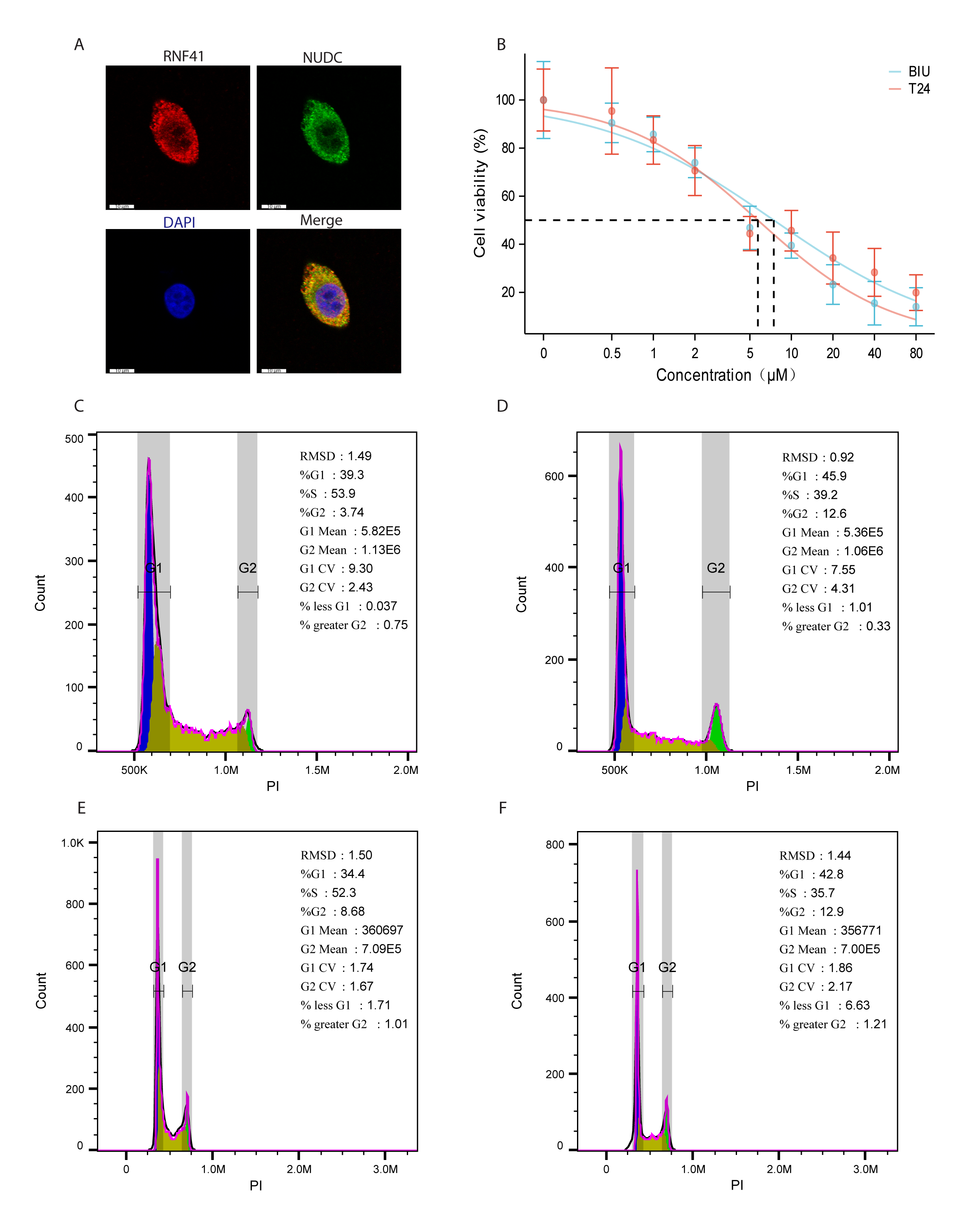


**Fig. S3**

(A) Fluorescence microscopy revealed co-localization of RNF41 (red) and NUDC (green) in BIU cells, with nuclear staining by 4', 6-diamidino-2-phenylindole (DAPI; blue). Scale bar, 10 μm. (B) The half maximal inhibitory concentration (IC50) of MMAE in T24 and BIU cells. (C-D) Flow cytometry was used to assess the cell cycle changes of untreated T24 cells (C) and T24 cells treated with 5μM MMAE (D). (E-F) Flow cytometry was used to assess the cell cycle changes of untreated BIU cells (E) and BIU cells treated with 5μM MMAE (F).

**Fig. S4**

**
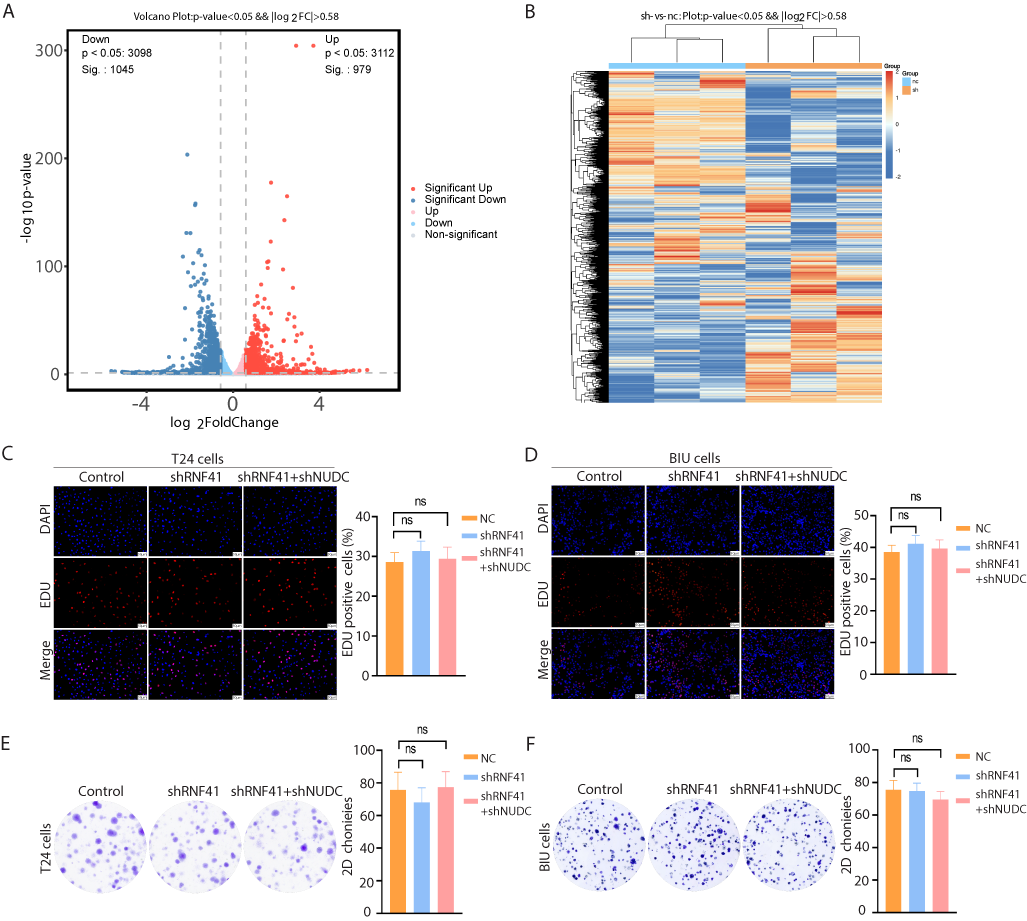
**

**Fig. S4**

(A) Volcano plot illustrating differentially expressed genes from RNA-seq analysis between RNF41 knockdown and control groups. (B) Cluster heatmap showing RNA-seq analysis of differentially expressed genes between the RNF41 knockout group and the control group. (C) (D) Representative images of EdU labeling in T24 and BIU cell rescue experiments (left) with corresponding quantification (right). (E) (F) Representative images of colony formation from T24 and BIU cell rescue experiments (left) with corresponding quantification (right).

**Supplementary Tables**

**Table S1. Clinicopathological variables and RNF41 expression in 34 bladder cancer patients sourced from the database platform.**

| **Variables** | **Total** | **RNF41** | | ***p* value** |
| --- | --- | --- | --- | --- |
|  | n=34 | Low (n=20) | High (n=14) |  |
| **Age** |  |  |  | 0.4090 |
| ≤65 | 15 | 10 | 5 |  |
| ＞65 | 19 | 10 | 9 |  |
| **Sex** |  |  |  | 0.8335 |
| Male | 32 | 19 | 13 |  |
| Female | 2 | 1 | 1 |  |
| **Tumor stage** |  |  |  | 0.0595 |
| Tis-T2 | 26 | 13 | 13 |  |
| T3-T4 | 8 | 7 | 1 |  |
| **Stage** |  |  |  | 0.0171***** |
| Ois-II | 24 | 11 | 13 |  |
| III-IV | 10 | 9 | 1 |  |
| **grade** |  |  |  | 0.0023****** |
| 1-2 | 8 | 1 | 7 |  |
| 3 | 26 | 19 | 7 |  |
| **Lymph node metastasis** |  |  |  | 0.0311***** |
| No | 28 | 14 | 14 |  |
| Yes | 6 | 6 | 0 |  |

^#^*p* value was analyzed by chi-sequared test; ^*^ indicates *p* < 0.05, ^**^*p* < 0.01 with statistical significance.

**Table S2 Clinicopathological variables and NUDC expression in 34 bladder cancer patients sourced from the database platform.**

| **Variables** | **Total** | **NUDC** | | **p value** |
| --- | --- | --- | --- | --- |
|  | n=34 | Low (n=12) | High (n=22) |  |
| **Age** |  |  |  | 0.6099 |
| ≤65 | 15 | 6 | 9 |  |
| ＞65 | 19 | 6 | 13 |  |
| **Tumor stage** |  |  |  | 0.0169***** |
| Tis-T2 | 26 | 12 | 14 |  |
| T3-T4 | 8 | 0 | 8 |  |
| **Stage** |  |  |  | 0.0464**^*^** |
| Ois-II | 24 | 11 | 13 |  |
| III-IV | 10 | 1 | 9 |  |
| **grade** |  |  |  | 0.0656 |
| 1-2 | 8 | 5 | 3 |  |
| 3 | 26 | 7 | 19 |  |
| **Lymph node metastasis** |  |  |  | 0.0770 |
| No | 28 | 12 | 16 |  |
| Yes | 6 | 0 | 6 |  |

#*p* value was analyzed by chi-sequared test; ^*^ indicates *p* < 0.05 with statistical significance.

**Supplementary Materials and Methods**

**Antibodies and reagents**

Anti-RNF41 antibody (Catalog No. 17233-1-AP), Anti-NUDC antibody (Catalog No. 10681-1-AP), anti-β-actin antibody (Catalog No. HRP-66009), anti-HA antibody (Catalog No. HRP-81290), anti-Flag antibody (Catalog No. 66008-4-Ig), and anti-Myc antibody (Catalog No. 16286-1-AP) were obtained from Proteintech (Wuhan, China). Anti-Ki67 antibody (ab15580) was acquired from Abcam (Cambridge, United Kingdom). All antibodies were utilized following the manufacturers’ guidelines.

**Plasmids and cloning**

Flag-RNF41 (G25067-9) and Myc-NUDC (P59968) expression vectors were constructed by subcloning into the pcDNA3 plasmid and were sourced from MiaoLingBio, China. Various domains of RNF41 were subcloned into the pcDNA3-Flag vector. The RNF41 mutant was produced using the QuikChange II Site-Directed Mutagenesis Kit. Primers utilized for cloning can be provided upon request.

**Stable transfection of cell lines**

The packaging and transduction of shRNA lentiviral vectors were conducted as previously (1). The lentiviral vectors shRNA-RNF41, shRNA-NUDC, and shRNA-GFP (control vector) were obtained from QEgene (Shanghai, China). In brief, 50 µL of PEI-MAX transfection reagent was combined with 5 µg of RNF41 or NUDC plasmid, along with equivalent amounts of two helper plasmids (PAX2 and MD2 at a 2:1 ratio), and then mixed with 1.5 mL of serum-free DMEM. After a 30-minute incubation, the plasmid mixture was introduced to cells in serum-free DMEM. Following 6 hours, 10% FBS was added, and the viral supernatant was collected 48 hours later. Stable transfectants were established following viral infection and puromycin selection.

**Quantitative real-time PCR (qRT-PCR)**

qRT-PCR was described as previously (1). Primers for qPCR analysis of human gene transcripts were:

RNF41:

Forward Primer: 5’-AGA CAC GCA TCG CAG AG-3’

Reverse Primer: 5’-GAA GGT TGG GGT TGA CAC T-3’

β-actin：

Forward Primer: 5’-TCT CCC AAG TCC ACA CAC GG-3’

Reverse Primer: 5’-GGC ACG AAG GCT CAT CA-3’

Primers for qPCR analysis of mouse gene transcripts were:

Western blotting

The cells were subjected to centrifugation and lysed on ice for 30 minutes using RIPA buffer containing protease and phosphatase inhibitors. Following this, the cell lysate was processed with an ultrasonic homogenizer for 10 seconds and centrifuged at 12,000 × g for 20 minutes. Protein separation and analysis were conducted as previously reported (2). Finally, protein samples were resolved using gradient SDS-PAGE and then transferred onto a PVDF membrane (Cytiva, catalog number: 10600021).

Cell proliferation and clony formation assays

Cell proliferation and clony formation were described as previously (3).

Wound healing

To assess the migration ability of BLCA cells, a wound healing assay was conducted using cells cultured in six-well plates. When the cells reached over 90% confluency, a straight scratch was made with a 200-μl pipette tip. The cells were then maintained in medium with reduced serum (3% FBS). The wells were imaged using a Celigo Imaging Cytometer at designated time points, and the images were analyzed with ImageJ software. The non-covered area of each well was calculated as an average percentage across three wells and presented as mean ± SD. This experiment was repeated three times for consistency.

Migration and invasion

Transwell assays were performed using 24-well plates equipped with 8 μm polycarbonate membrane filters (Corning Inc., Corning, NY), either coated with or without Matrigel. T24 and BIU cells (20,000 per well) were seeded in serum-free DMEM or 1640 media in the upper chamber, while 700 µl of medium supplemented with 10% FBS was added to the lower chamber as a chemoattractant. After incubation for 24–48 hours, cells were fixed with 4% paraformaldehyde for 15 minutes and stained with 0.1% crystal violet for 10 minutes. Cells that had migrated to the underside of the filter were visualized and imaged under a light microscope. Cell counts were taken from five random fields per insert, with each data point representing the mean from three wells.

**EDU assay**

Transfected cells were seeded at a density of 5,000 cells per well in a 96-well plate. After 24 hours, the culture medium was replaced with a 1:1,000 dilution of EdU and incubated for an additional 2 hours. Staining was performed following the protocol provided in the EdU assay kit (C10310-1, RiboBio). Imaging was carried out using a confocal microscope.

Protein immunoprecipitation (IP) and Liquid Chromatography-MS Analysis

Co-immunoprecipitation (Co-IP) was conducted as previously (2). In brief, cells were collected in RIPA lysis buffer, incubated on ice for 30 minutes, and then centrifuged at 12,000×g for 20 minutes. Cell lysates were immunoprecipitated with specific antibodies and incubated overnight at 4 °C with Flag-M2 beads or protein A/G agarose beads (Santa Cruz, USA), rotating gently. The beads were washed three times with lysis buffer. The eluted proteins were then separated using SDS-PAGE and analyzed by Western blotting. For Liquid Chromatography-Mass Spectrometry (LC-MS) analysis, immunoprecipitation with RNF41 antibodies was performed as described. The precipitated proteins were eluted three times with lysis buffer, subjected to in-solution trypsin digestion, and then analyzed by LC-MS. Protein identification was conducted using the Mascot (v2.3.02) program, comparing results against the Uniprot human protein database (released Dec 2014).

Ubiquitination assay

Ubiquitination assays were performed according to established protocols (1). Cells were transfected with the specified plasmids and lysed using immunoprecipitation buffer. During the immunoprecipitation process, 2 mg of protein was mixed with the designated antibodies and incubated overnight at 4 °C, after which Flag-M2 beads or protein A/G beads were added for 2 hours. The beads were washed once with TBS containing 1% Triton X-100 and 1% SDS, followed by two washes with 0.5 M LiCl and TBS buffer, and finally washed in PBS with 1% Triton X-100. The ubiquitination of the specified antibodies was then assessed via immunoblotting.

Immunofluorescence

**Cells were cultured on cover slips, fixed in 4% paraformaldehyde in PBS for 30 minutes, and then permeabilized with 0.1% Triton X-100 in PBS for 5 minutes. The cover slips were blocked in PBS containing 2% BSA and 1% normal goat serum for 1 hour at room temperature. Next, the slips were incubated with primary antibodies in the blocking solution overnight at 4 °C in a humid chamber. After washing three times with PBS, they were treated with Alexa Fluor Cy3-conjugated and/or Alexa Fluor 488-conjugated secondary antibodies in the blocking solution for 1 hour at 37 °C. The slips were then washed again three times with PBS and counterstained with 4,6-diamidino-2-phenylindole (DAPI) for nuclear visualization. Images were captured using an inverted microscope with a ×40 objective and scanned with a laser confocal system.**

Supplemental References

1. Xu S, Fan L, Jeon HY, Zhang F, Cui X, Mickle MB, et al. p300-Mediated Acetylation of Histone Demethylase JMJD1A Prevents Its Degradation by Ubiquitin Ligase STUB1 and Enhances Its Activity in Prostate Cancer. Cancer Res. 2020 Aug 1;80(15):3074–87.

2. Xu SH, Huang JZ, Xu ML, Yu G, Yin XF, Chen D, et al. ACK1 promotes gastric cancer epithelial-mesenchymal transition and metastasis through AKT-POU2F1-ECD signalling. J Pathol. 2015 Jun;236(2):175–85.

3. Tang DE, Dai Y, He JX, Lin LW, Leng QX, Geng XY, et al. Targeting the KDM4B-AR-c-Myc axis promotes sensitivity to androgen receptor-targeted therapy in advanced prostate cancer. J Pathol. 2020 Oct;252(2):101–13.
