# Supplementary material for: Loss of RNF41 promotes bladder cancer metastasis through increasing NUDC stability to enhance tubulin polymerization
Source: Cell Death Dis. 2025 Jun 10;16(1):443. doi: 10.1038/s41419-025-07758-y (PMC12152122; doi:10.1038/s41419-025-07758-y)
Supplement: Supplementary file 2 — original western blots [file 41419_2025_7758_MOESM2_ESM.pdf]

Western Blot and PCR Raw data

Figure 1

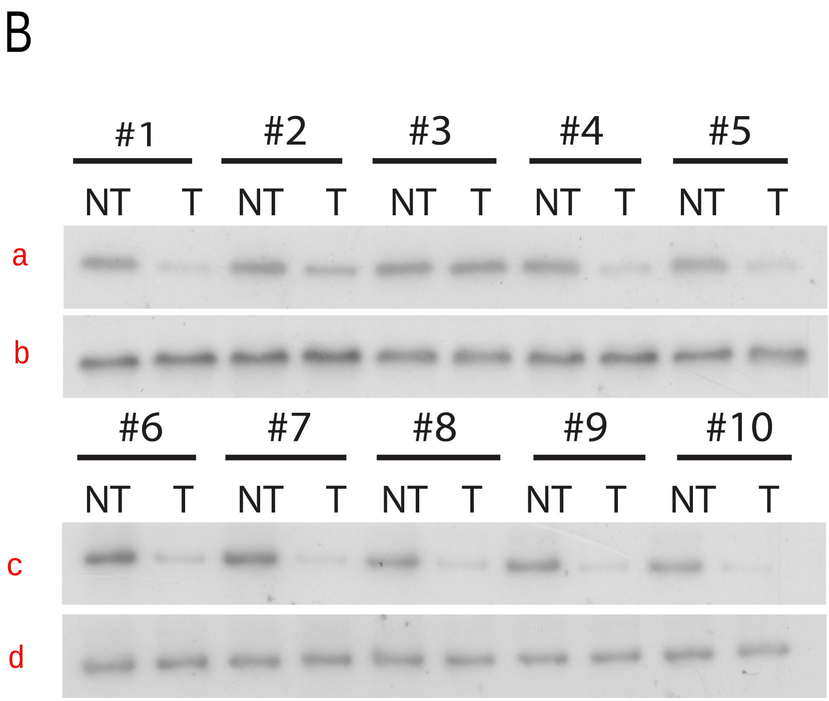

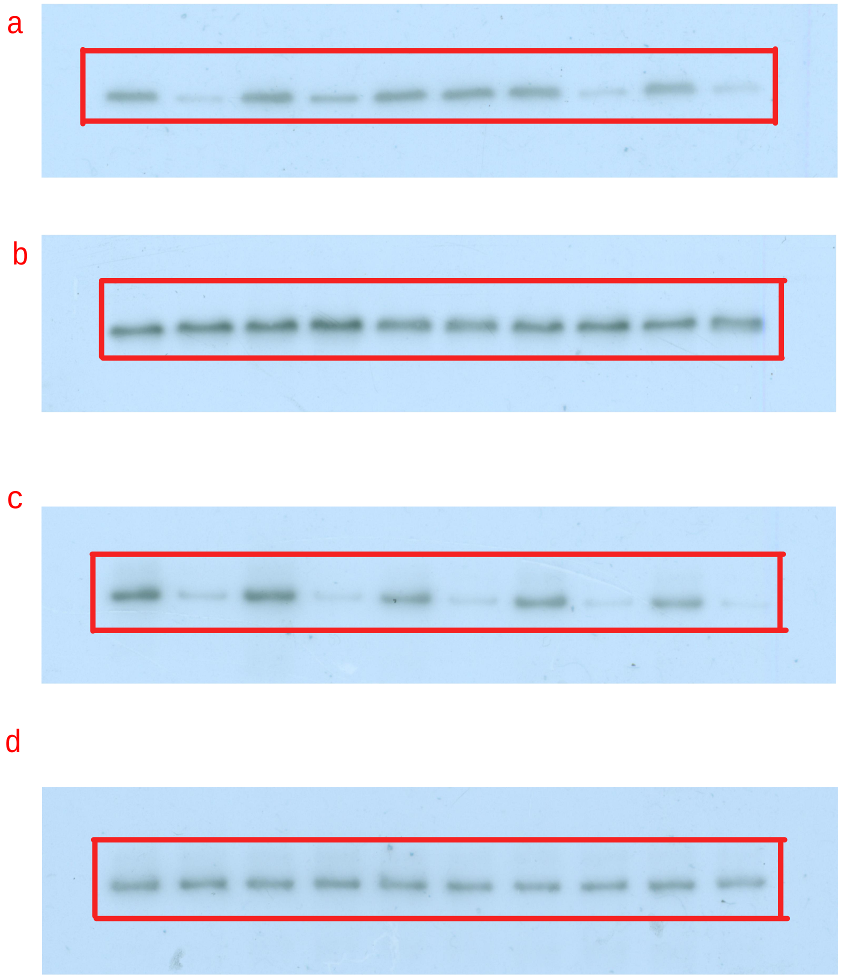

C

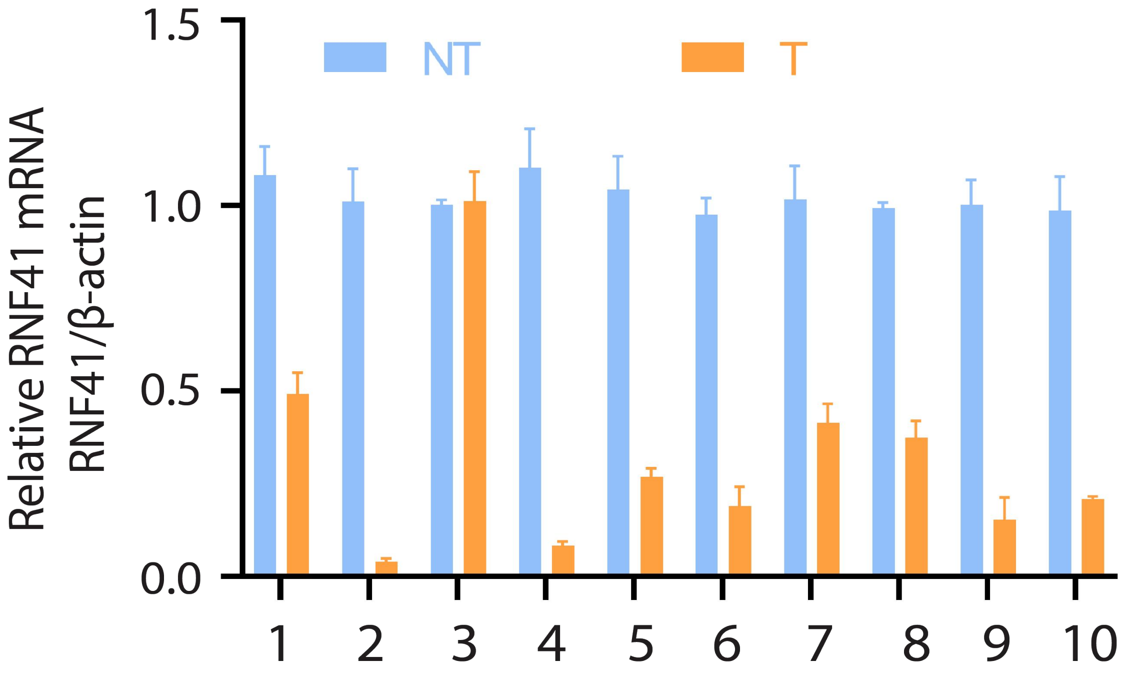

	NT			T		
1	1.138963293	1	1.111344897	0.433483107	0.51469512	0.533114694
2	0.934214942	1.104503725	1	0.034345316	0.048915088	0.039184878
3	0.989874293	1	1.016889059	1.068309214	1.044913199	0.928302428
4	1.196968147	1.118184888	1	0.096569428	0.077978879	0.076255107
5	1	1.147096372	0.991135361	0.280875576	0.24581211	0.28262742
6	1.003183597	0.92717029	1	0.20482441	0.146506475	0.231994163
7	1	1.114426522	0.942856447	0.431394252	0.363374199	0.453895968
8	1	1.00334514	0.976886473	0.415686613	0.380063132	0.33205638
9	1	1.070441212	0.93979574	0.180741246	0.104325491	0.188971056
10	0.898211115	1.070053517	1	0.203357926	0.215694274	0.207918611

Figure 2

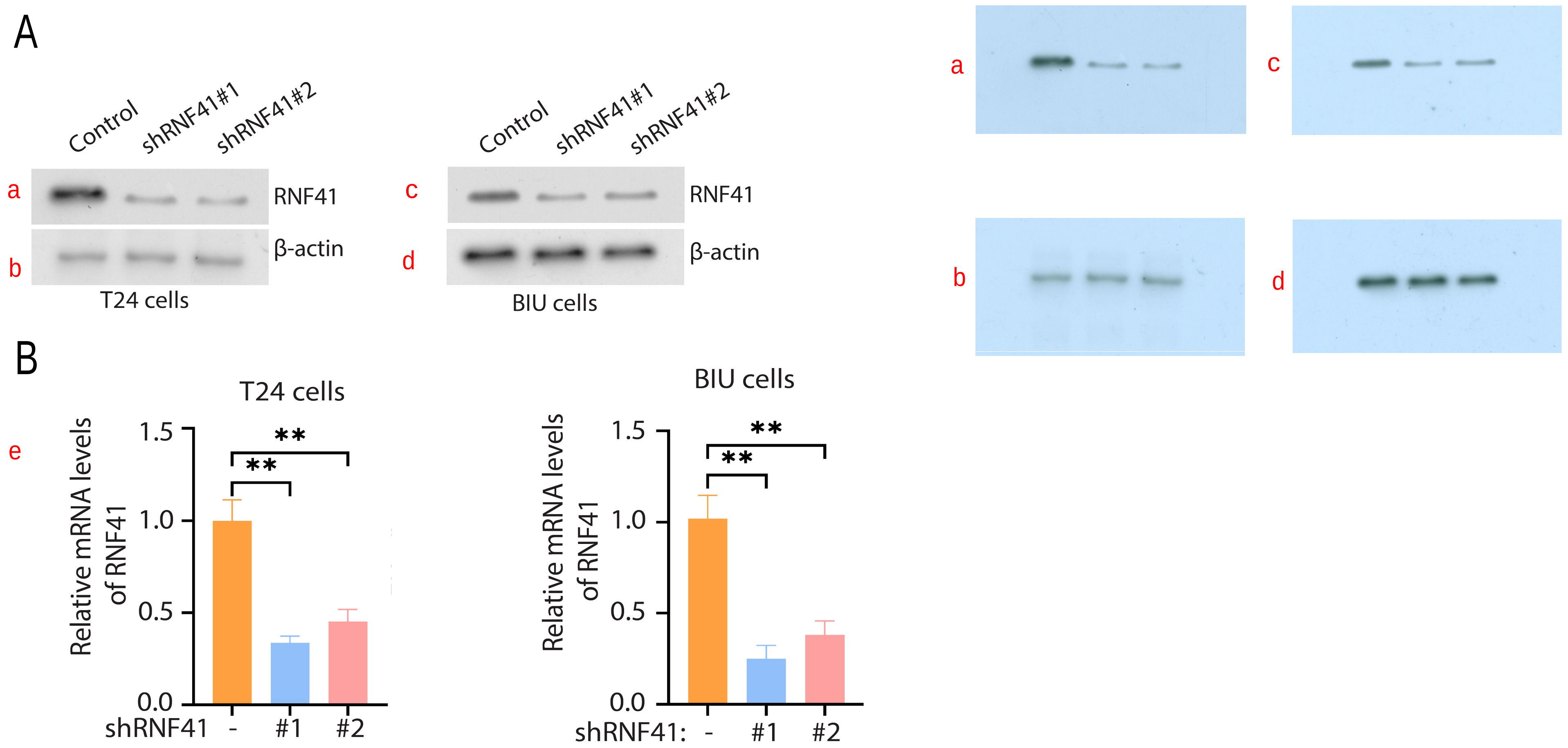

e

	NC			SH1			SH2		
T24	0.886630668	1	1.11306156	0.350518947	0.366872067	0.298639919	0.441386643	0.523981107	0.392389594
BIU	0.903075571	1	1.155473882	0.227808887	0.332770046	0.186918296	0.424759862	0.294598388	0.427061144

Figure 3

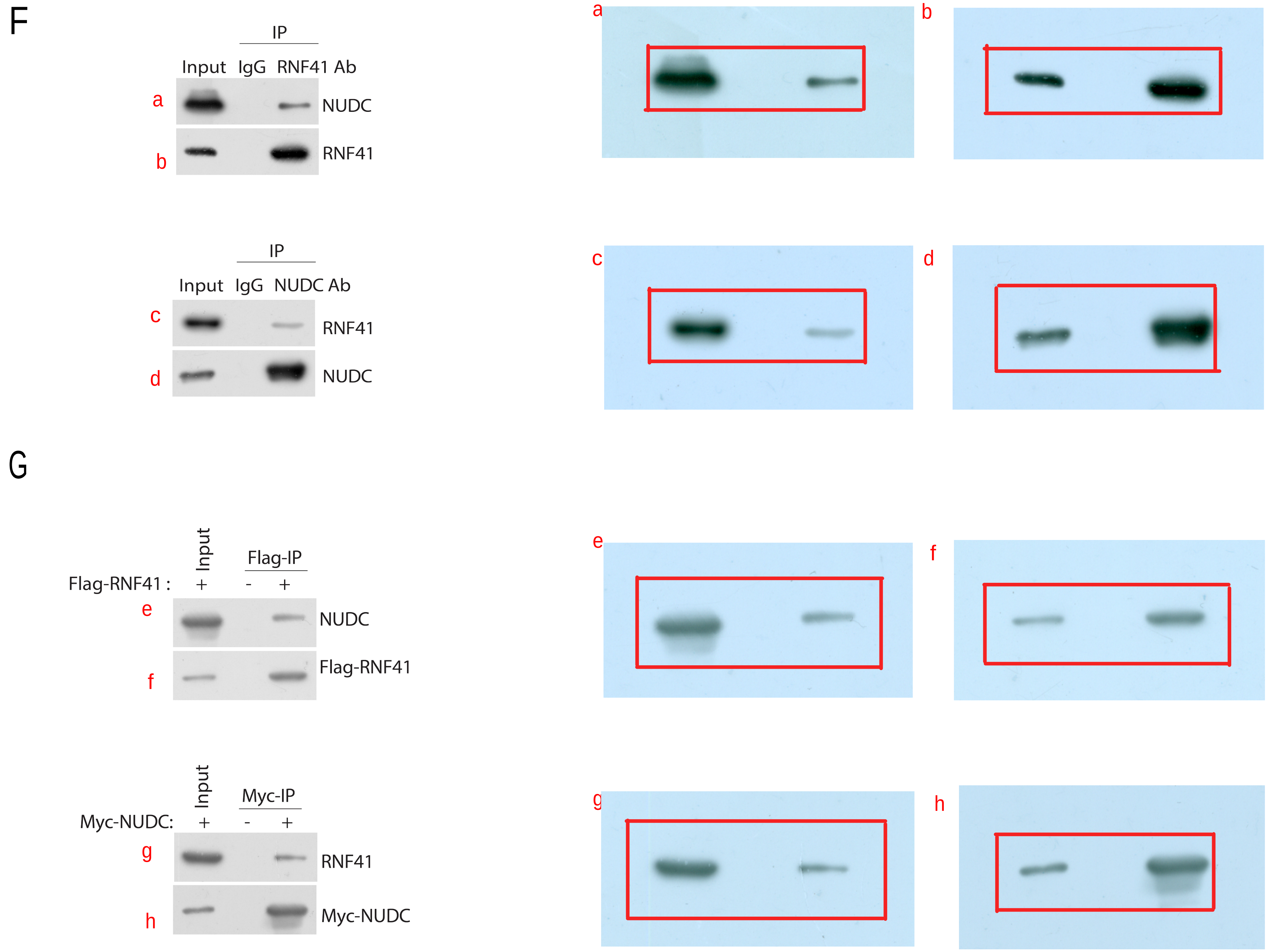

F
IP
Input IgG RNF41 Ab
a NUDC
b RNF41
IP
Input IgG NUDC Ab
c RNF41
d NUDC
G
Input
Flag-IP
Flag-RNF41 : + - +
e NUDC
f Flag-RNF41
Input
Myc-IP
Myc-NUDC: + - +
g RNF41
h Myc-NUDC
a
b
c
d
e
f
g
h

Figure 3

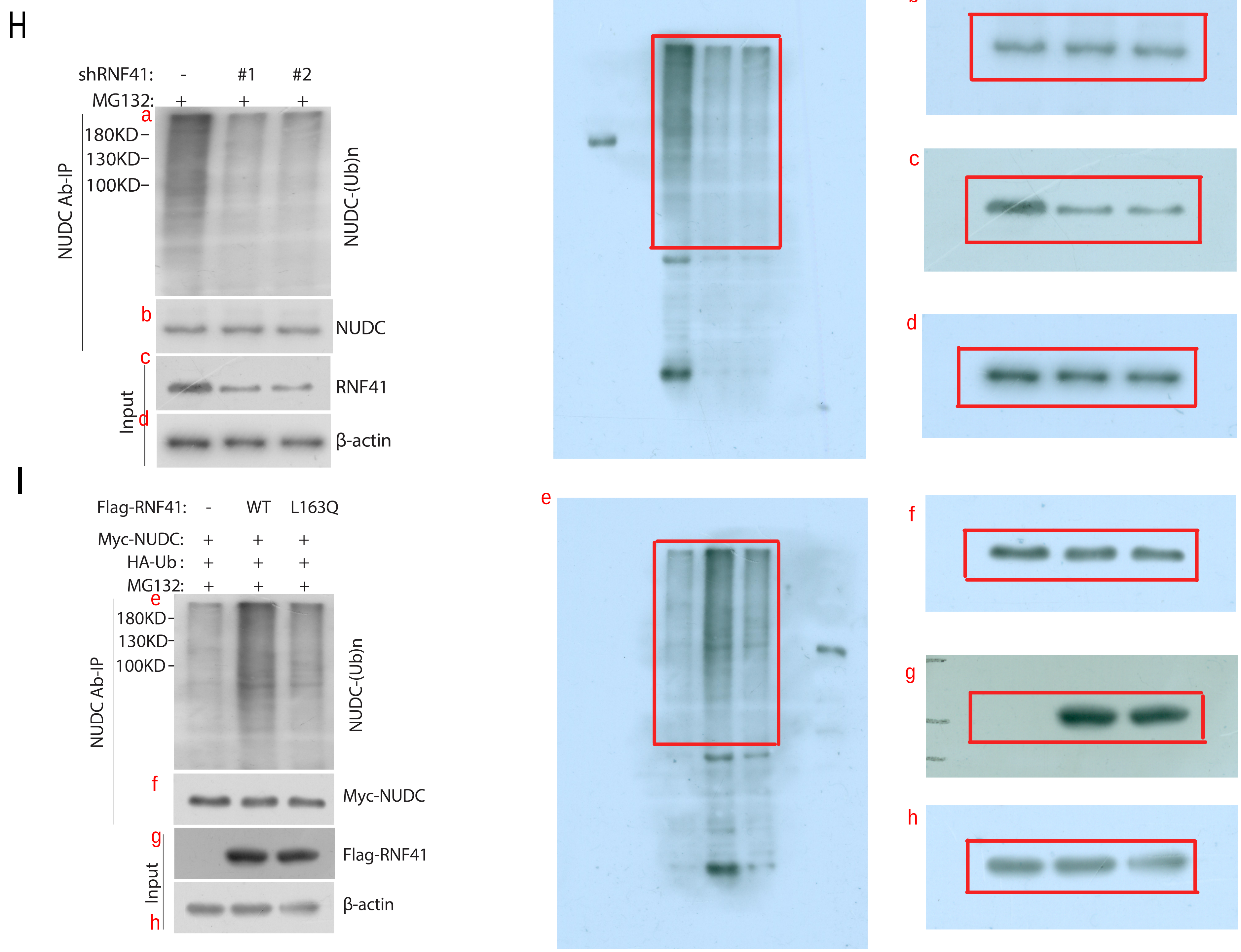
H
shRNF41: - #1 #2
MG132: + + +
a
180KD
130KD
100KD
NUDC Ab-IP
NUDC-(Ub)n
b
NUDC
c
RNF41
Input
d
β-actin
I
Flag-RNF41: - WT L163Q
Myc-NUDC: + + +
HA-Ub: + + +
MG132: + + +
e
180KD
130KD
100KD
NUDC Ab-IP
NUDC-(Ub)n
f
Myc-NUDC
g
Flag-RNF41
Input
h
β-actin
a
b
c
d
e
f
g
h

Figure 3

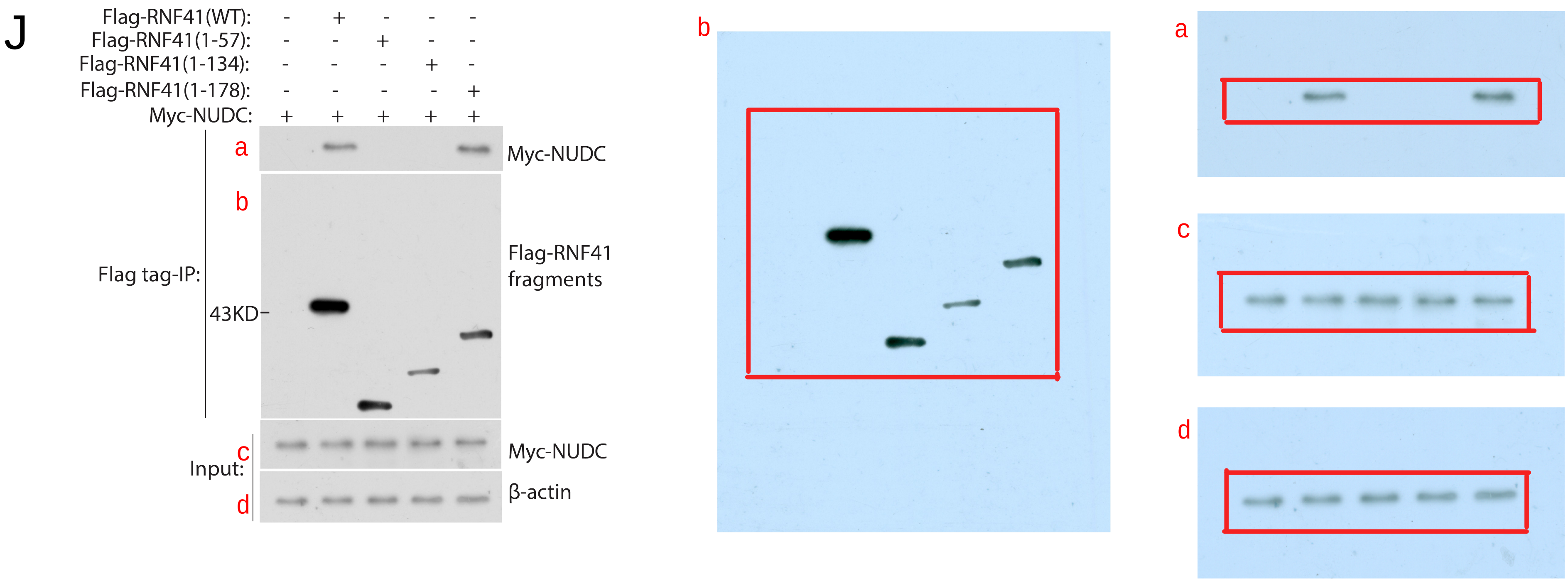

J
Flag-RNF41(WT): - + - - -
Flag-RNF41(1-57): - - + - -
Flag-RNF41(1-134): - - - + -
Flag-RNF41(1-178): - - - - +
Myc-NUDC: + + + + +
a
Myc-NUDC
b
Flag tag-IP:
Flag-RNF41 fragments
43KD
c
Input:
Myc-NUDC
d
β-actin
b
a
c
d

Figure 4

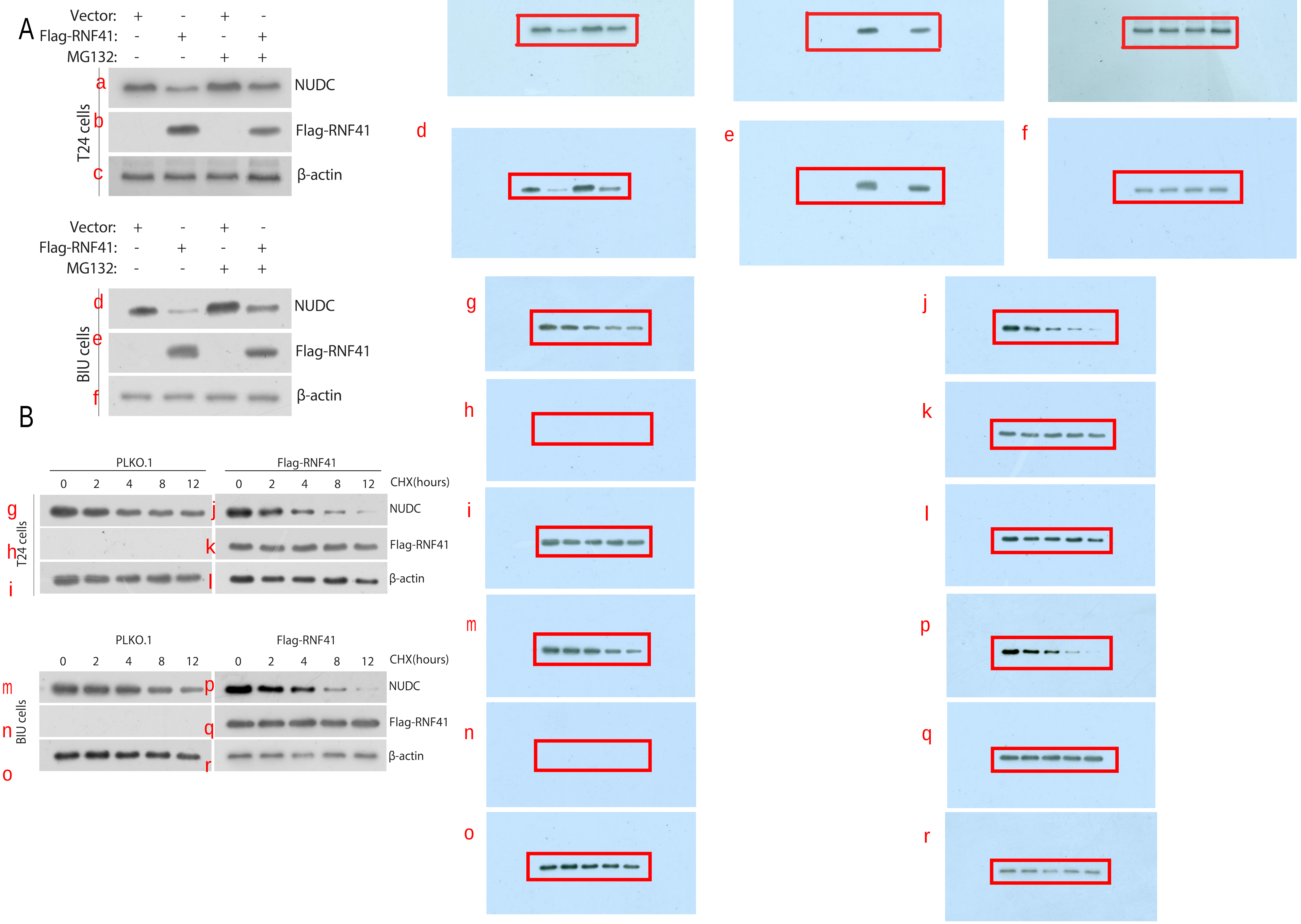

A
Vector: + - + -
Flag-RNF41: - + - +
MG132: - - + +
T24 cells
NUDC
Flag-RNF41
β-actin
BIU cells
B
PLKO.1
Flag-RNF41
0 2 4 8 12
CHX(hours)
a
b
c
d
e
f
g
h
i
j
k
l
m
n
o
p
q
r

Figure 4

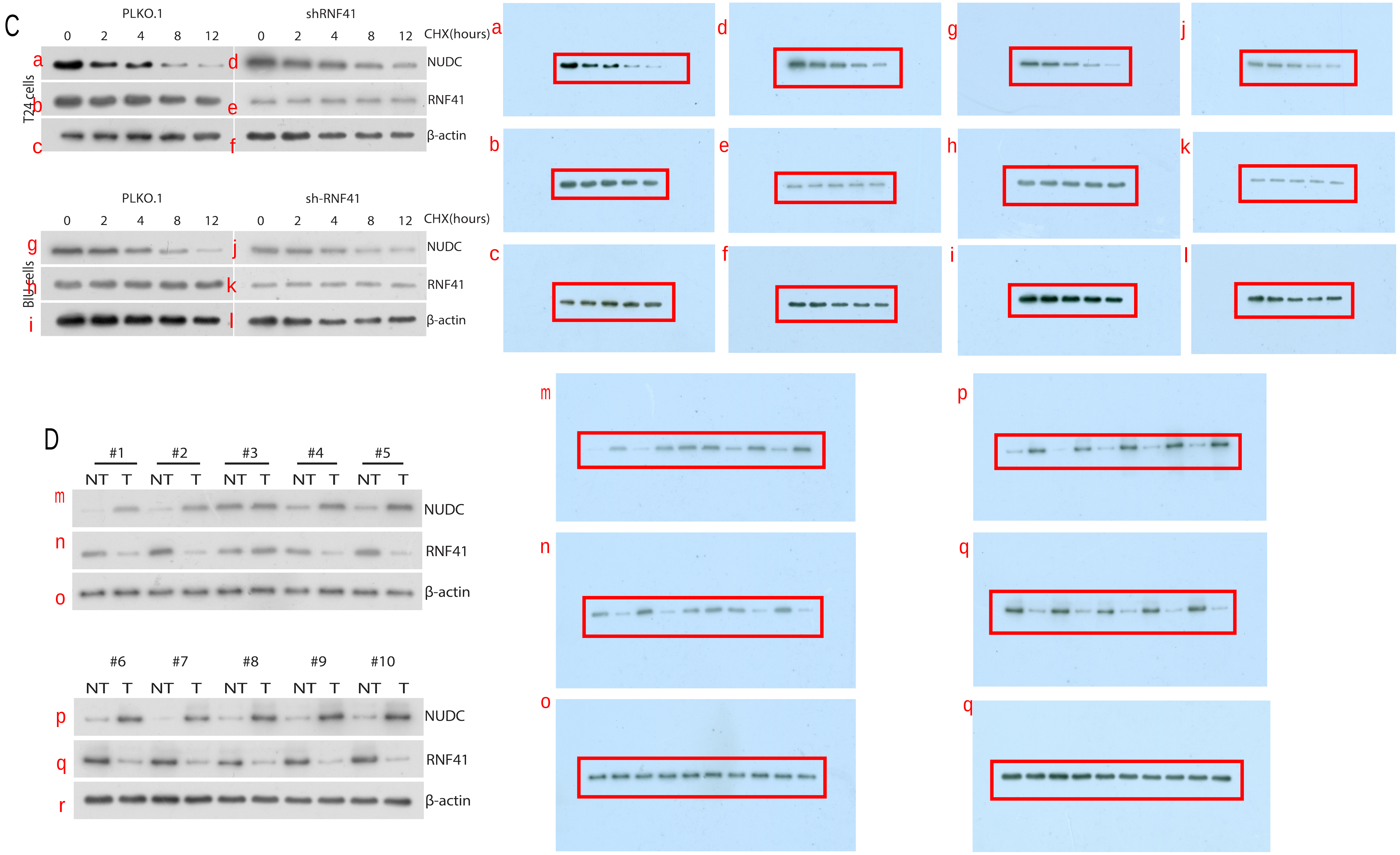
C
PLKO.1
shRNF41
0 2 4 8 12 0 2 4 8 12 CHX(hours)
NUDC
RNF41
β-actin
T24 cells
PLKO.1
sh-RNF41
0 2 4 8 12 0 2 4 8 12 CHX(hours)
NUDC
RNF41
β-actin
BIU cells
a b c d e f g h i j k l
D
#1 #2 #3 #4 #5
NT T NT T NT T NT T NT T
NUDC
RNF41
β-actin
#6 #7 #8 #9 #10
NT T NT T NT T NT T NT T
NUDC
RNF41
β-actin
m n o p q r

Figure 5

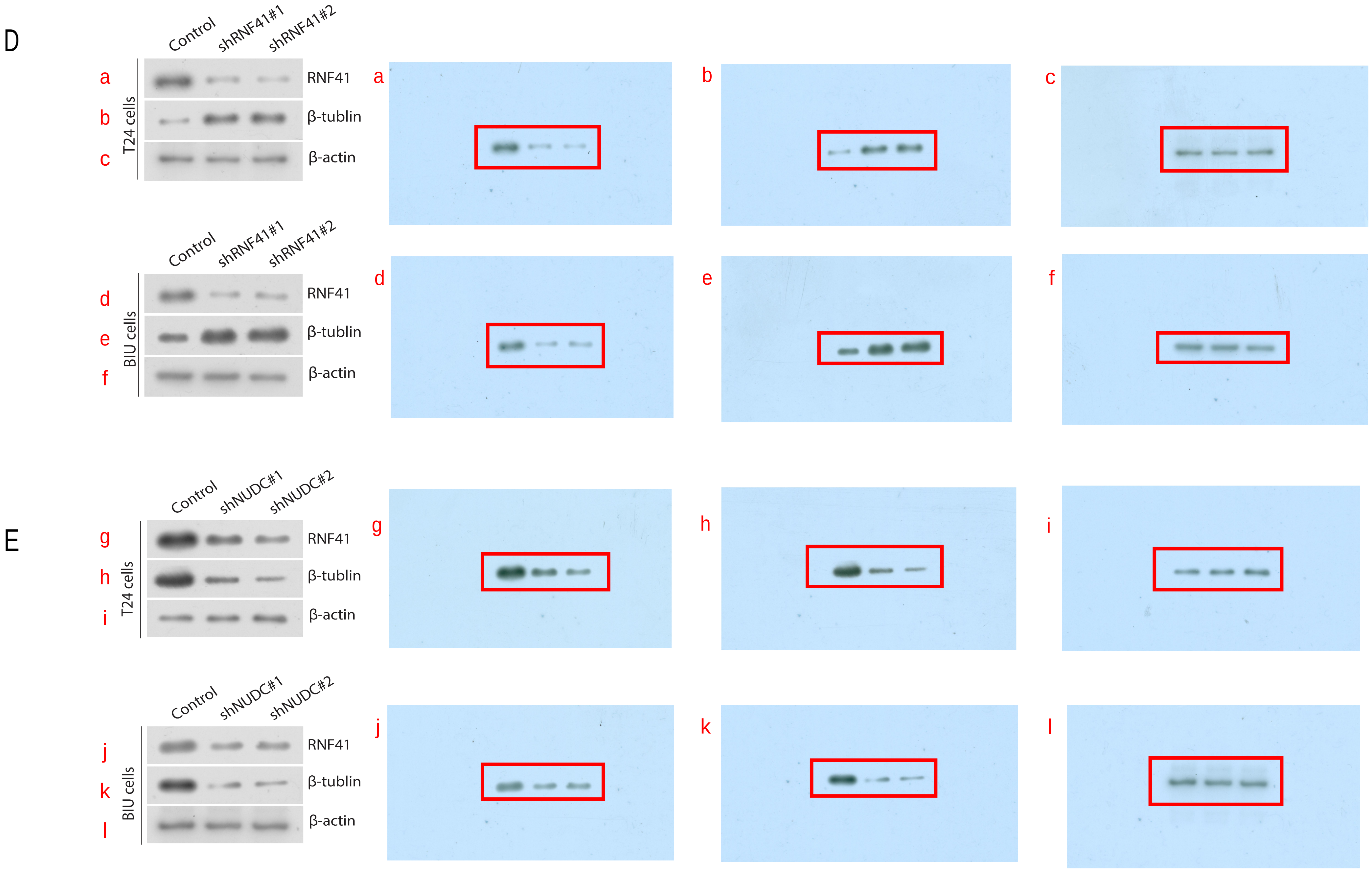
D
Control
shRNF41#1
shRNF41#2
a
b
c
T24 cells
RNF41
β-tublin
β-actin
d
e
f
BIU cells
E
Control
shNUDC#1
shNUDC#2
g
h
i
j
k
l

Figure 5

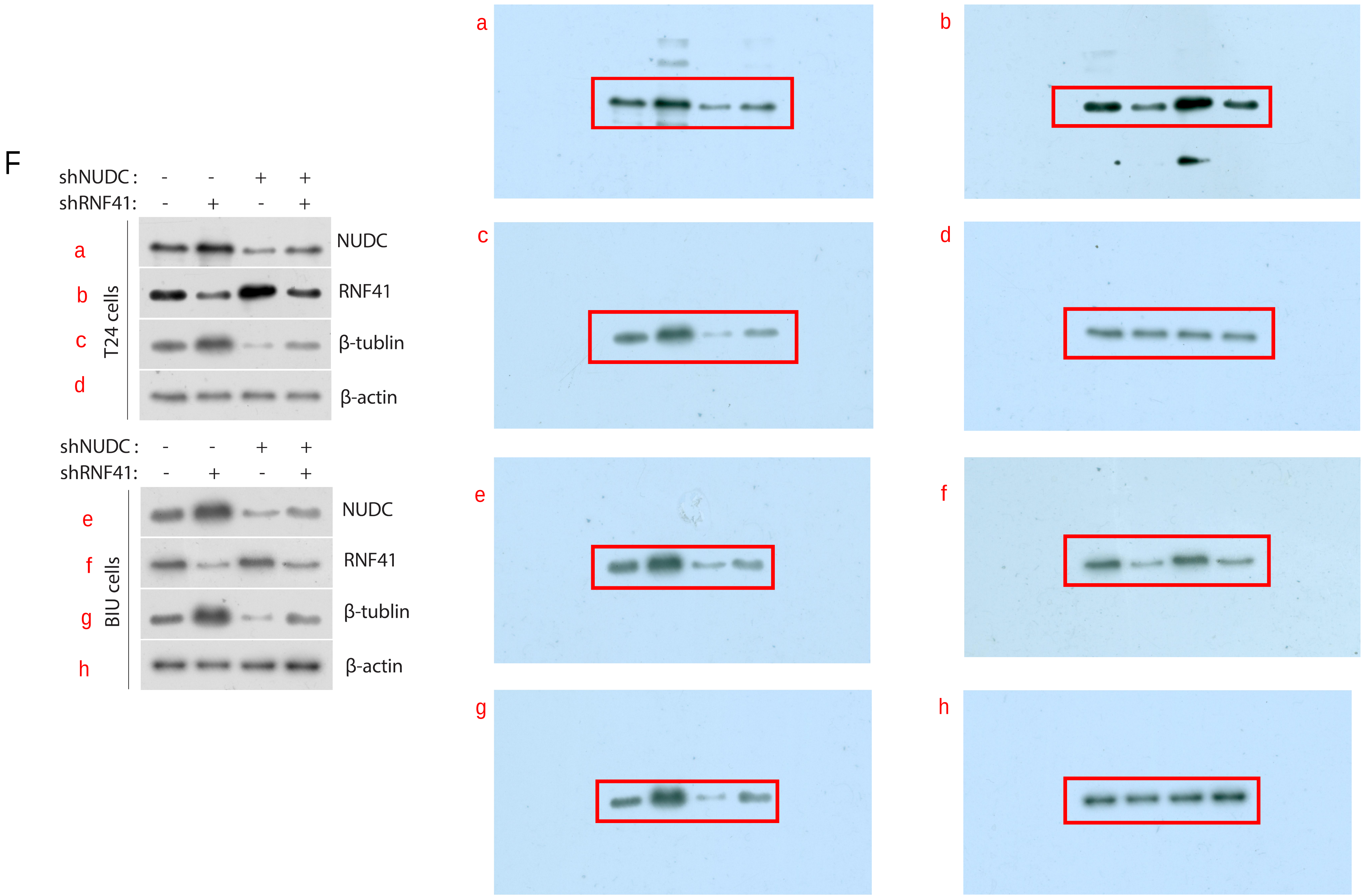
F
shNUDC : - - + +
shRNF41: - + - +
a
b
c
d
T24 cells
NUDC
RNF41
β-tublin
β-actin
shNUDC : - - + +
shRNF41: - + - +
e
f
g
h
BIU cells
NUDC
RNF41
β-tublin
β-actin
a
b
c
d
e
f
g
h

Figure 5

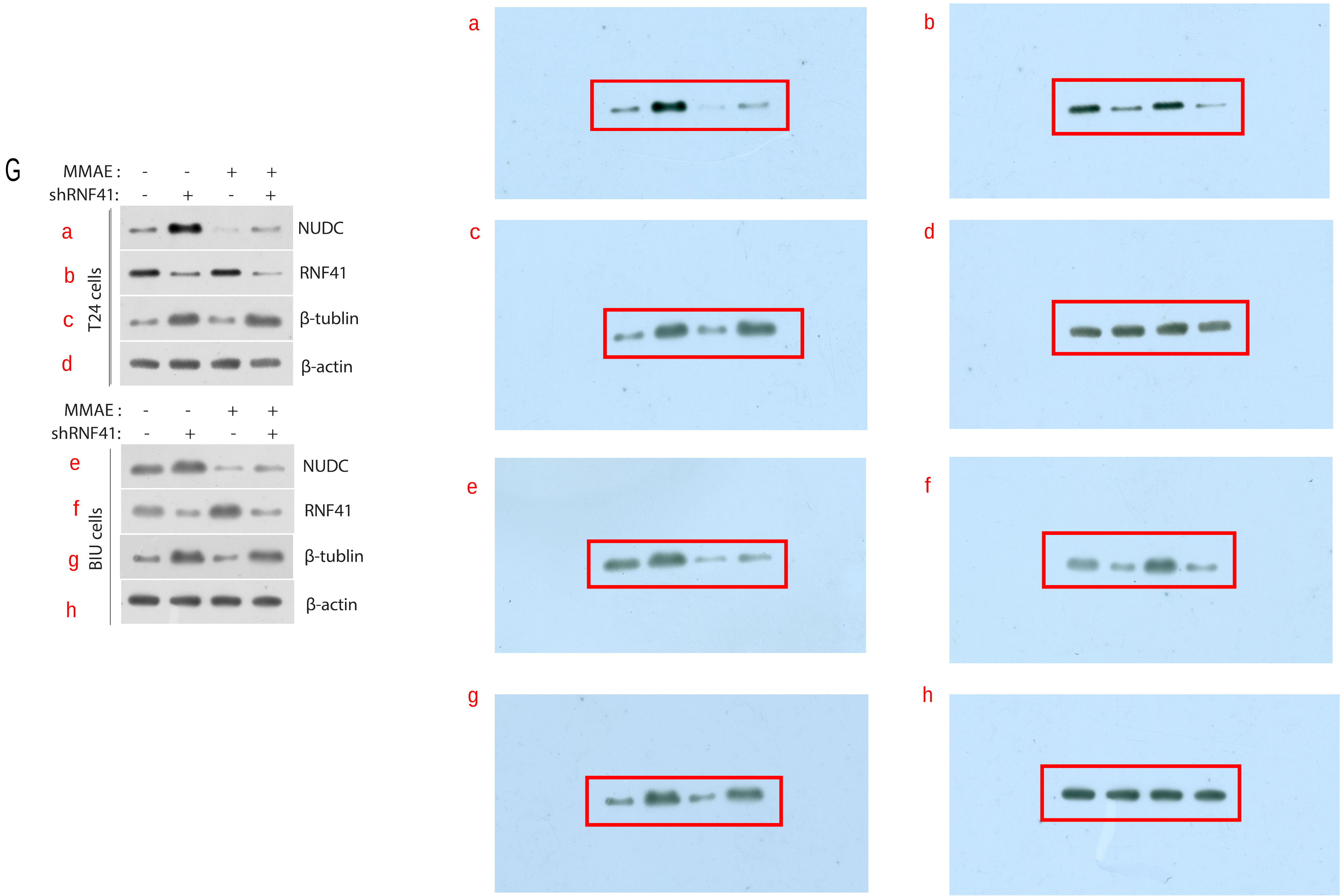
G
MMAE : - - + +
shRNF41: - + - +
a
b
c
d
T24 cells
NUDC
RNF41
β-tublin
β-actin
MMAE : - - + +
shRNF41: - + - +
e
f
g
h
BIU cells
NUDC
RNF41
β-tublin
β-actin
a
b
c
d
e
f
g
h

Figure 6

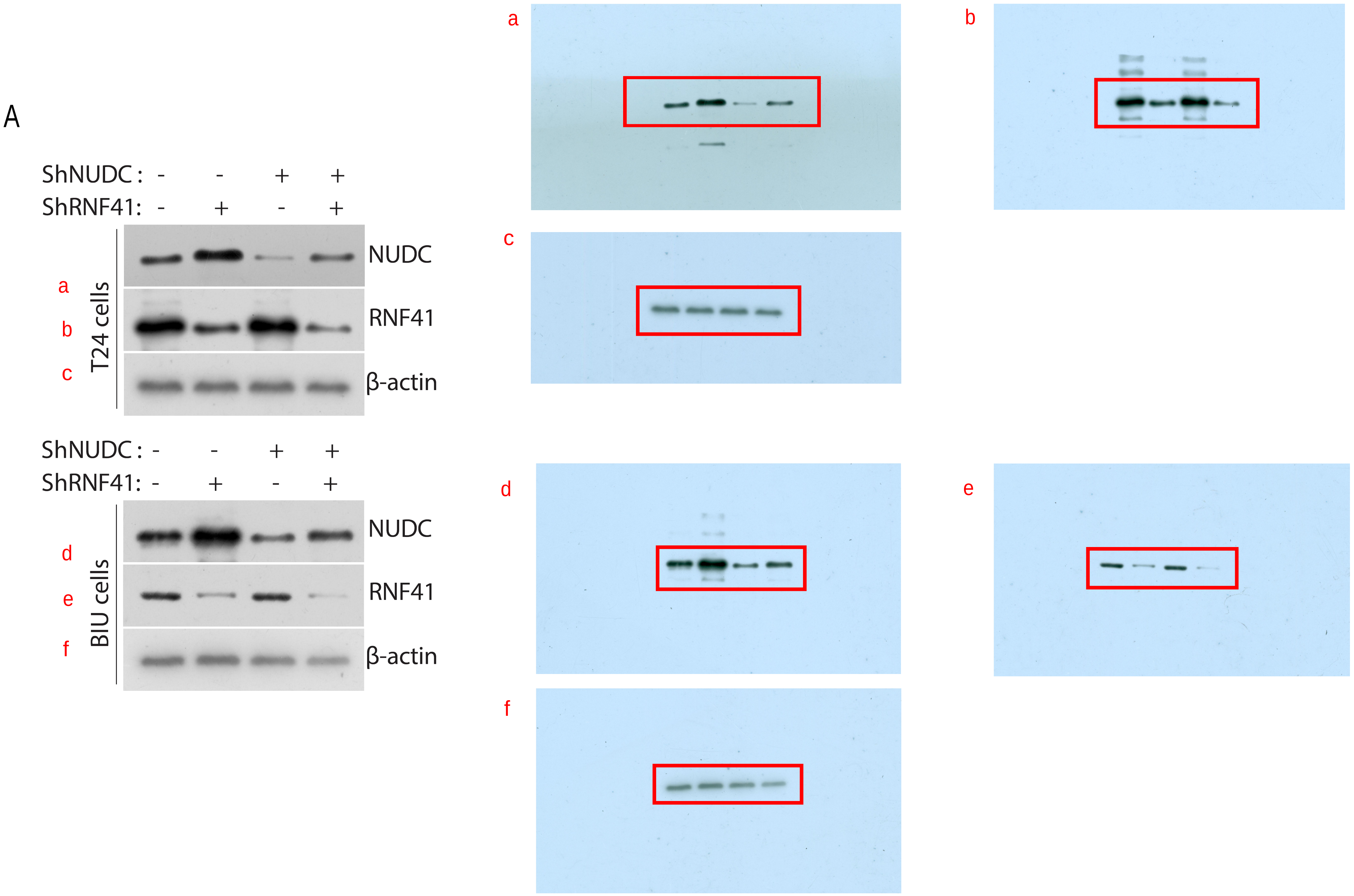
A
ShNUDC: - - + +
ShRNF41: - + - +
a
b
c
T24 cells
NUDC
RNF41
β-actin
ShNUDC: - - + +
ShRNF41: - + - +
d
e
f
BIU cells
NUDC
RNF41
β-actin
a
b
c
d
e
f

Supplement data Figure

Figure S2 A

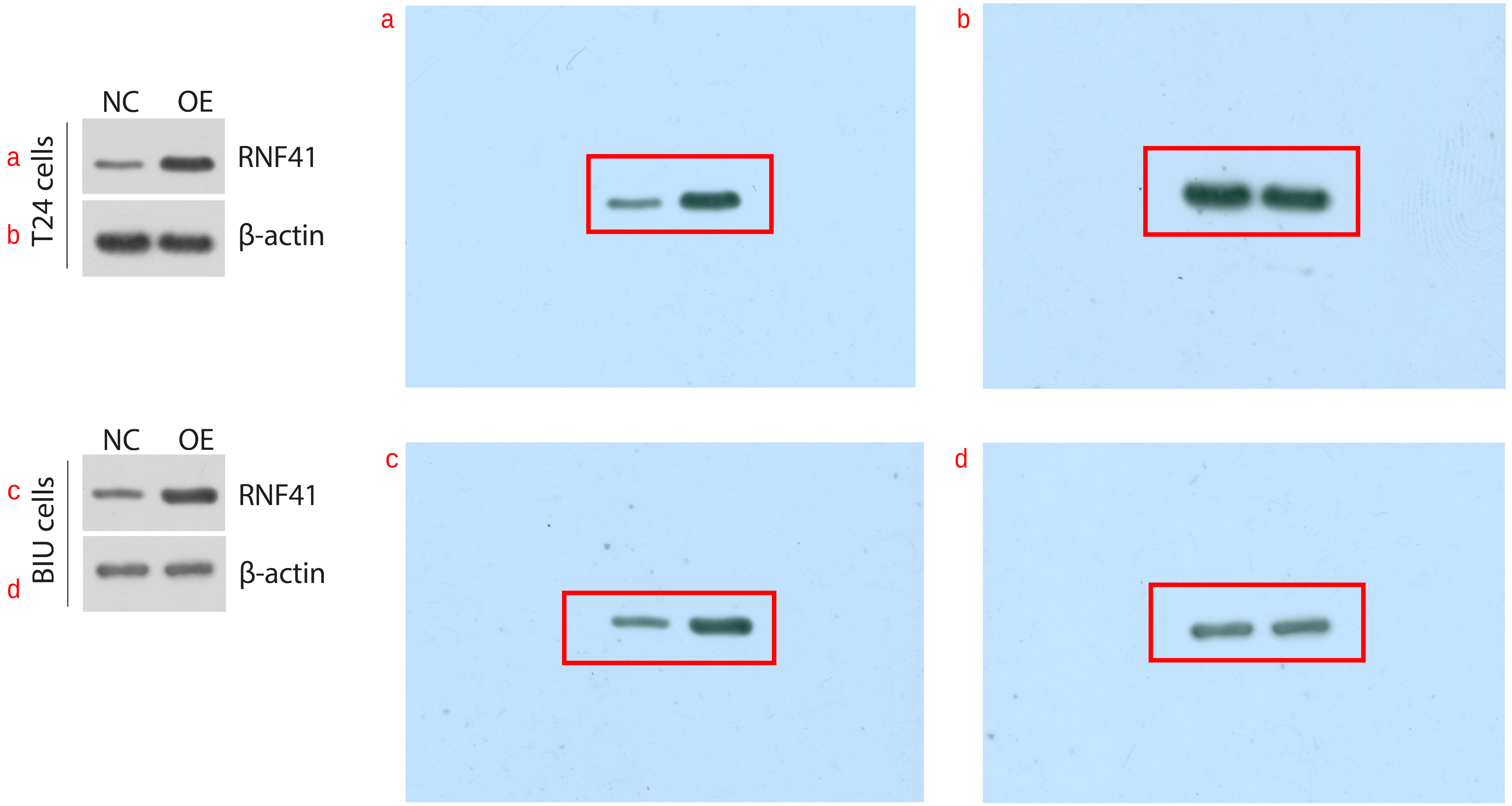

Figure S2 B

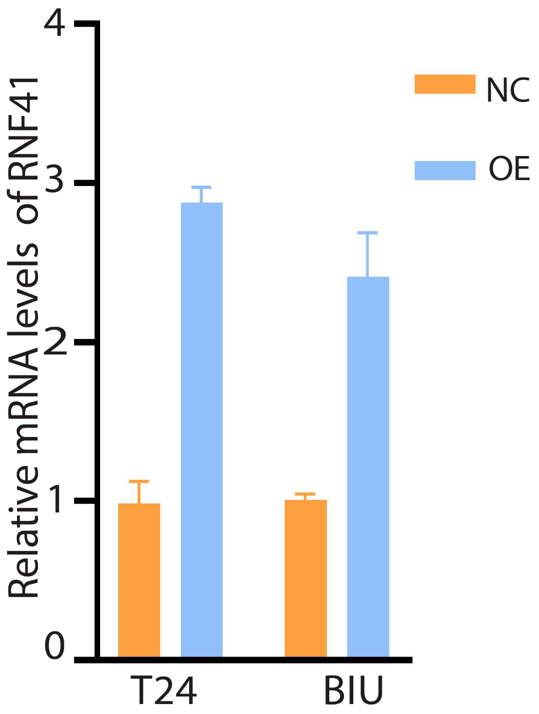

	NC			OE		
T24	0.854522003	1	1.113404484	2.830123446	2.820967551	2.989332746
BIU	1.047334923	0.980863374	1	2.685102761	2.416721556	2.162257494
